# Universal Hamiltonian simulators in one and two dimensions

**DOI:** 10.1038/s41467-026-71686-4

**Published:** 2026-04-21

**Authors:** Leo Zhou, Dorit Aharonov

**Affiliations:** 1 Electrical and Computer Engineering Department, University of California, Los Angeles, CA USA; 2 Walter Burke Institute for Theoretical Physics, Caltech, Pasadena, CA USA; 3 Institute for Quantum Information and Matter, Caltech, Pasadena, CA USA; 4 School of Computer Science and Engineering, The Hebrew University, Jerusalem, Israel

**Keywords:** Quantum simulation, Computer science, Information theory and computation, Condensed-matter physics

## Abstract

A major quantum computing application is analog Hamiltonian simulations, in which the low-lying spectrum of a simulator Hamiltonian ${H}^{{\prime} }$ encodes the physics of a target Hamiltonian *H*. Some families of 2D spin-lattice Hamiltonians—such as the Heisenberg or XY model on the square lattice—are known to be “universal” simulators, in the sense that they can simulate any target Hamiltonian. Unfortunately, while the known simulations of 1D or 2D target Hamiltonians are efficient, they require resources growing exponentially with system sizes for target Hamiltonians with general connectivity. This leaves many important cases such as 3D or all-to-all-connected target Hamiltonians out of reach in practice. Here, we remedy this situation and show how the known 2D universal families of Hamiltonians and a new 1D family can simulate all target local Hamiltonians, with only polynomial-scaling overhead. This exponential improvement is achieved by a nonperturbative method combining the quantum phase-estimation algorithm and the circuit-to-Hamiltonian construction. Furthermore, all Hamiltonians efficiently simulable by quantum circuits, including nonlocal ones, also have efficient analog simulators in our 2D and 1D universal families. Our work establishes that analog simulations of general Hamiltonians can be made efficient, significantly expanding the application potential of analog Hamiltonian simulations in near-term quantum technologies.

## Abstract (web summary)

It is known that some 2D spin-lattice Hamiltonians are “universal” simulators, in the sense that they can simulate any target Hamiltonian, but only at the cost of an exponential overhead. Here, the authors demonstrate that this can actually be done with polynomial-scaling.

## Introduction

Analog Hamiltonian simulations enable reproducing the physics of a given target Hamiltonian *H* by another, easier to implement Hamiltonian ${H}^{{\prime} }$. Such simulations have a vast range of possible applications, e.g., probing new many-body physics, developing new materials and drugs^1^, as well as implementing Hamiltonian-based quantum algorithms^2–4^. Indeed, simulating many-body quantum physics was the major motivation in Feynman’s 1981 proposal^5^ for quantum computers. Cirac and Zoller^6^ further argued for the importance of analog simulations based on their less stringent requirements on error correction and controls compared to fault tolerant quantum computers. This robustness suggests that such simulations could already be useful in noisy quantum devices^7^. Exciting demonstrations of this approach have been observed in recent years, where coherent analog Hamiltonian simulations in systems as large as thousands of quantum particles^8^ have been successfully realized to address many-body physics questions^9–16^.

For analog Hamiltonian simulators to be practical for a wide range of applications, an important consideration is that the simulator should be easy to implement and not be re-engineered on a case-by-case basis. Furthermore, it is often highly desirable for the simulator to be embeddable in a simple architecture with easy physical realization, e.g., a low-dimensional lattice.

An important step towards such a “universal Hamiltonian simulator" theory was taken by Cubitt, Montanaro, and Piddock in ref. ^17^. There, they took inspiration from the notion of *a universal set of quantum gates*; namely, a (usually finite) set of quantum gates that can implement any general quantum computation. Replacing gates by Hamiltonian terms, ref. ^17^ precisely defined analog Hamiltonian simulation, and what it means for a family of Hamiltonians to be *universal*. According to their definition, for any target local Hamiltonian *H*, there should exist a Hamiltonian ${H}^{{\prime} }$ in the family that can simulate *H*. The ability to implement these universal families thus enables analog simulation of all local Hamiltonians. Ref. ^17^ proves that various simple families of quantum spin-lattice models in two dimensions with tunable nearest-neighbor interaction energy are universal. More families were shown to be universal in this sense by later works^18–20^. Importantly, it was shown in ref. ^17^ that analog simulations can reproduce all physical properties of the target system—including time-evolution, thermal states, and effects of local noise processes—to any desired precision.

Nevertheless, a problem in the constructions given in all above-mentioned works^17–20^ is that they are not efficient for general target Hamiltonians. The efficiency in fact depends on the connectivity, or spatial dimensionality, of the target. Any target Hamiltonian *H* set on a 1D or 2D lattice can be simulated by a universal Hamiltonian simulator set on a 2D lattice, incurring only a polynomial overhead in both the number of particles and the interaction energy. However, if the target *H* is embedded in a higher dimension (e.g., when *H* is 3D or has all-to-all interactions), an exponential overhead is required, either in the interaction energy or the number of particles simulating the interactions using gadgets^21^, which also gives up spatial locality.

We call such families, which can reproduce the physics but not guarantee the efficiency in simulating general local Hamiltonians, *weakly universal*. Recall that the whole point behind analog Hamiltonian simulations (which also originally motivated Feynman) is to avoid the exponential overhead that one incurs in classical simulations of many-body quantum systems.

In this work, we overcome the problem of exponential overhead and arrive at what we call *strongly universal* Hamiltonian simulators, which guarantee efficient simulation of all target local Hamiltonians, regardless of their geometry. We believe this is perhaps a more suitable definition of universality. Specifically, we provide a construction of analog simulators that requires only polynomial overhead in both the number of particles and the interaction energy, and enables simulation in 2D of any target local Hamiltonian regardless of its connectivity (Theorem 1). In fact, the construction can be done using 2D spin-lattice models which include only a single type of nearest-neighbor interaction, where spatial variation appears solely in the interaction energies—a property we call *semi-translation-invariance*. As a consequence, the semi-translation-invariant 2D families of Hamiltonians found to be weakly universal in ref. ^17^ are in fact also strongly universal when more efficient constructions are utilized. We further demonstrate that strong universality is achievable in 1D (Theorem 2), albeit at the cost of spatially varying interaction types; while not semi-translation-invariant, this constructed simulator remains efficient. The key technical advance enabling these results is a non-perturbative Hamiltonian-circuit-Hamiltonian mapping that efficiently simulates all-to-all interactions with spatially local couplings. Table 1 compares the resources required for simulating general Hamiltonians by our constructions to previous Hamiltonian simulator constructions.

**Table 1:** The properties of currently known constructions of universal families of Hamiltonians that can simulate any local *n*-qudit Hamiltonian

	spatial dimension	interaction energy	particle number	translation-invariance
Cubitt et al.^17^	2D	exp(poly(*n*))	poly(*n*)	semi
Piddock-Bausch^19^	2D	exp(poly(*n*))	exp(poly(*n*))	full
Kohler et al.^20^	1D	exp(poly(*n*))	poly(*n*)	full*
Kohler et al.^20^	1D	exp(poly(*n*))	exp(poly(*n*))	full
This work (Theorem 1)	2D	poly(*n*)	poly(*n*)	semi
This work (Theorem 2)	1D	poly(*n*)	poly(*n*)	none

Note the 1D construction by Kohler et al. ^20^ with full* translation-invariance uses a Hamiltonian interaction that changes depending on the target Hamiltonian.

Our work significantly expands the applicability domain of simple analog Hamiltonian simulators. In particular, we show that the aforementioned families of 1D and 2D universal analog simulators can efficiently simulate any Hamiltonian that has an efficient digital quantum simulation, i.e., any Hamiltonian whose time-evolution *e*^−*iHt*^ has a polynomial-sized circuit approximation. This includes fermionic Hamiltonians as well as Hamiltonians with poly(*n*) many $O(\log n)$-local terms which are digitally simulable via Trotter decomposition and the Solovay-Kitaev algorithm^22^. Both classes are beyond the class of *O*(1)-local Hamiltonians targeted by the original definition of universal simulators in ref. ^17^. More generally, since any physically realizable physical system can be simulated using a quantum circuit by the quantum Church-Turing thesis^23,24^, our results shows that our analog 1D and 2D simulators can efficiently simulate any physically realizable quantum system. Finally, we clarify the relationship between analog simulation and Hamiltonian dynamics simulation, which only simulates the evolution *e*^−*iHt*^ applied to any input state. We show that while analog simulation implies dynamics simulation, the converse does not hold; for completeness, we also present universality results for dynamics simulation.

## Results

### Theory of analog Hamiltonian simulation

We start by describing the well-motivated definition of ref. ^17^ for analog simulation, which posits that “${H}^{{\prime} }$ simulates *H*” if the full spectrum of *H* can be encoded as the low-lying part of the spectrum of ${H}^{{\prime} }$, by an encoding that preserves locality of observables. More precisely:

#### Definition 1

(Local encoding, adapted from ref. ^17^): Consider an encoding map ${{\mathcal{E}}}:{\mbox{Herm}}({({{\mathbb{C}}}^{d})}^{\otimes n})\to {\mbox{Herm}}({({{\mathbb{C}}}^{{d}^{{\prime} }})}^{\otimes {n}^{{\prime} }})$ that takes Hermitian operators on *n* qudits (*d*-dimensional particles) into operators acting on ${n}^{{\prime} }\,{d}^{{\prime} }$-dimensional particles (where ${d}^{n}\le {{d}^{{\prime} }}^{{n}^{{\prime} }}$). We say ${{\mathcal{E}}}$ is an *η*-*local encoding* if we can write

1 $${{\mathcal{E}}}(A)=V(A\otimes P+\bar{A}\otimes Q){V}^{{\dagger} },$$

such that *V* is an isometry satisfying ∥*V* − *V*_loc_∥ ≤ *η* for some local isometry ${V}_{loc}{=\bigotimes }_{i=1}^{n}{V}_{i}$, where each *V*_*i*_ is an isometry acting on one qudit in the original system. *P* and *Q* are locally orthogonal projectors, i.e., *P* and *Q* are orthogonal projectors and  ∀ *i* ∃ orthogonal projectors *P*_*i*_, *Q*_*i*_ acting on the same subsystem as *V*_*i*_ such that *P*_*i*_*Q*_*i*_ = 0, *P*_*i*_*P* = *P* and *Q*_*i*_*Q* = *Q* (see also [^17^, Supporting Information, Theorem 15]). $\bar{A}$ is the complex conjugation of *A*. ∥ ⋅ ∥ is the spectral norm.

When *η* = 0, we say ${{\mathcal{E}}}$ is a *local encoding*.

Furthermore, ${{{\mathcal{E}}}}^{state}$ is an *η*-*local state-encoding* if it is of the form ${{{\mathcal{E}}}}^{state}(\rho )={{{\mathcal{E}}}}^{{\prime} }(\rho )/Tr({{{\mathcal{E}}}}^{{\prime} }(\rho ))$ for some *η*-local encoding ${{{\mathcal{E}}}}^{{\prime} }$. For a given local encoding, one natural choice of the corresponding local state-encoding would set ${{{\mathcal{E}}}}^{{\prime} }={{\mathcal{E}}}$. An alternative choice of local state-encoding could take ${V}^{{\prime} }=V$, ${P}^{{\prime} }=\sigma$, and ${Q}^{{\prime} }=0$ where *σ* is a state satisfying *Pσ* = *σ*.

#### Definition 2

(Analog Hamiltonian simulation, adapted from ref. ^17^): A Hamiltonian ${H}^{{\prime} }$ is a (Δ, *η*, *ϵ*)-*simulation* of an *n*-qudit Hamiltonian *H* if there exists an *η*-local encoding ${{{\mathcal{E}}}}_{\eta }$ as in Definition 1 such that

1. $${{{\mathcal{E}}}}_{\eta }({\mathbb{1}})={P}_{\le \Delta ({H}^{{\prime} })}$$;
2. $$\parallel {H}_{\le \Delta }^{{\prime} }-{{{\mathcal{E}}}}_{\eta }(H)\parallel \le \epsilon$$.

Here, ${P}_{\le \Delta ({H}^{{\prime} })}$ is the projector onto the subspace of eigenstates of ${H}^{{\prime} }$ with eigenvalue ≤Δ, and ${H}_{\le \Delta }^{{\prime} }={P}_{\le \Delta ({H}^{{\prime} })}{H}^{{\prime} }$ is the restriction of ${H}^{{\prime} }$ onto these states. We say the simulation is *efficient* if both the number of particles in ${H}^{{\prime} }$ and its maximum energy $\parallel {H}^{{\prime} }\parallel$ are at most *O*(poly(*n*, *η*^−1^, *ϵ*^−1^, Δ)), and the description of ${H}^{{\prime} }$ is efficiently computable.

Under this definition, ref. ^17^ showed that implementing ${H}^{{\prime} }$ allows one to approximately reproduce all physical properties of *H*, implying that the term “simulation” means essentially reproducing any physical aspect. Specifically, since ${{{\mathcal{E}}}}_{\eta }$ is an *η*-local encoding, all local observables *A* with respect to *H* are mapped to local observables ${{{\mathcal{E}}}}_{0}(A)$ for ${H}^{{\prime} }$ up to error *η*. Correspondingly, there is a local state-encoding ${{{\mathcal{E}}}}_{0}^{state}(\rho )={{{\mathcal{E}}}}_{0}(\rho )/Tr[{{{\mathcal{E}}}}_{0}(\rho )]$ that satisfies $Tr(A\rho )=Tr[{{{\mathcal{E}}}}_{0}(A){{{\mathcal{E}}}}_{0}^{state}(\rho )]$. Gibbs states (thermal ensembles) of *H* are mapped to Gibbs states of ${H}^{{\prime} }$, with errors exponentially suppressed by the energy cutoff Δ. Time-evolution *e*^−*iHt*^ applied to any initial state *ρ* can also be simulated by ${e}^{-i{H}^{{\prime} }t}$ applied on the appropriately encoded state ${{{\mathcal{E}}}}_{0}^{state}(\rho )$, and the error in this simulation grows as *O*(*ϵt* + *η*).

Additionally, it has been argued that such analog simulators may not require active error-correction^6^. This is made more explicit in ref. ^17^, which argued that this is indeed true under Definition 2 and a reasonable assumption that the noise causes no significant leakage into the high energy subspace separated by a sufficiently large Δ. Then any local noise channel ${{{\mathcal{N}}}}^{{\prime} }$ acting on the simulator system can be approximated as an encoded version of a local noise channel ${{\mathcal{N}}}$ in the original system: $\parallel {{{\mathcal{N}}}}^{{\prime} }({{{\mathcal{E}}}}_{0}^{state}(\rho ))-{{{\mathcal{E}}}}_{0}^{state}({{\mathcal{N}}}(\rho )){\parallel }_{1}\le O(\eta )$ (see [^17^, Corollary 33 in SI]). Even when the encoded noise process is not the same as the true one, the noisy simulation by ${H}^{{\prime} }$ may still allow one to probe, without error-correction, the relevant physics of *H* that are insensitive to details of the noise processes.

Our goal is to understand which families of Hamiltonians can be used to simulate all other physical Hamiltonians as in Definition 2, with only polynomial overhead in all resources, for all target local Hamiltonians. To this end, we define:

#### Definition 3

(Weak and strong universality): A family of Hamiltonians ${{\mathcal{F}}}=\{{H}_{m}^{{\prime} }\}$ is *weakly universal* if given any Δ, *η*, *ϵ* > 0, any *O*(1)-local *n*-particle Hamiltonian *H* can be (Δ, *η*, *ϵ*)-simulated by some ${H}_{m}^{{\prime} }\in {{\mathcal{F}}}$. Such a family is *strongly universal* if for all *H* with ∥*H*∥≤poly(*n*), the simulation is always efficient —i.e., ${H}_{m}^{{\prime} }$ is efficiently computable in *O*(poly(*n*, *η*^−1^, *ϵ*^−1^, Δ)) time from the description of *H*, requires *O*(poly(*n*, *η*^−1^, *ϵ*^−1^, Δ)) particles, and $\parallel {H}_{m}^{{\prime} }\parallel=O({{\mbox{poly}}}(n,{\eta }^{-1},{\epsilon }^{-1},\Delta ))$.

We remark that this notion of strong universality is equivalent to the “efficient universality” used in prior works (e.g., ^17^). We adopt the “strong/weak” terminology here to emphasize that some universal families lack a known polynomial bound on resources. Note that weak universality does not mean that the simulations are provably inefficient.

Following ref. ^17^, we consider Hamiltonian simulators that only involve up to 2-local interactions drawn from a set ${{\mathcal{S}}}={\{{h}_{\alpha }\}}_{\alpha }$. Note this set ${{\mathcal{S}}}$ may contain only a single term. Then we define an ${{\mathcal{S}}}$-Hamiltonian simulator to be a Hamiltonian of the following form:

2 $${H}^{{\prime} }={\sum }_{\langle i,j\rangle \in E}{J}_{ij}{h}_{{\alpha }_{ij}}^{(i,j)}, {{\mbox{where}}}\,\parallel {h}_{{\alpha }_{ij}}\parallel \le 1 \,{{\mbox{and}}}\; {J}_{ij}\in {\mathbb{R}}.$$

Here, *E* is some set of edges describing the connectivity of the qudits, ${h}_{{\alpha }_{ij}}^{(i.j)}$ is some two-body operator ${h}_{{\alpha }_{ij}}\in {{\mathcal{S}}}$ acting on qudit *i* and *j*, and *J*_*ij*_ is the interaction energy.

It is shown in ref. ^17^ that many families of ${{\mathcal{S}}}$-Hamiltonians on qubits are weakly universal, even when restricting the connectivity *E* of the Hamiltonian simulator to the 2D square lattice. Two examples are the Heisenberg interaction (${{\mathcal{S}}}=\{X\otimes X+Y\otimes Y+Z\otimes Z\}$) and the XY-interaction (${{\mathcal{S}}}=\{X\otimes X+Y\otimes Y\}$). Here and below, we denote (*X*, *Y*, *Z*) = (*σ*_*x*_, *σ*_*y*_, *σ*_*z*_) as the Pauli matrices. With these choices of ${{\mathcal{S}}}$, the Hermitian terms in Eq. (2) are all equal up to their relative weights. To state this more concisely, we define:

#### Definition 4

(Full and Semi-Translation-Invariance): A Hamiltonian ${H}^{{\prime} }$ has *semi-translation-invariance (or semi-TI)* if all two-body operators are the same up to the scaling by *J*_*ij*_, i.e., ${h}_{{\alpha }_{ij}}^{(i,j)}={h}^{(i,j)}$. We say ${H}^{{\prime} }$ has *full translation-invariance (or full-TI)* if it has semi-translation-invariance and all the interaction energies are the same, i.e., *J*_*ij*_ = *J*.

Ref. ^17^ shows that any family of ${{\mathcal{S}}}$-Hamiltonians is weakly universal as long as ${{\mathcal{S}}}$ is non-2SLD, which means that the 2-local parts of all the interactions in ${{\mathcal{S}}}$ are not simultaneously and locally diagonalizable:

#### Definition 5

(2SLD interactions^17^): Suppose ${{\mathcal{S}}}$ is a set of interactions on 2 qubits. We say ${{\mathcal{S}}}$ is *2SLD* if there exists *U* ∈ SU(2) such that for each ${H}_{i}\in {{\mathcal{S}}}$, ${U}^{\otimes 2}{H}_{i}{({U}^{{\dagger} })}^{\otimes 2}={\alpha }_{i}Z\otimes Z+{A}_{i}\otimes {\mathbb{1}}+{\mathbb{1}}\otimes {B}_{i}$, where ${\alpha }_{i}\in {\mathbb{R}}$ and *A*_*i*_, *B*_*i*_ are 1-local operators. Otherwise, ${{\mathcal{S}}}$ is *non-2SLD*.

The main result of ref. ^17^ is then:

#### Theorem CMP18

(^17^): Any ${{\mathcal{S}}}$-Hamiltonian family set on a 2*D* square lattice of qubits is weakly universal as long as ${{\mathcal{S}}}$ is non-2SLD.

There are known non-2SLD sets of interactions ${{\mathcal{S}}}$ which contain a single interaction term (e.g., Heisenberg or XY interactions), thus semi-translation-invariant families of Hamiltonians in 2D that are weakly universal exist. Later works have extended this result to show weak universality of various families that use qudits^18^, are embedded in higher dimensions^19^, or are fully translation-invariant in 2D or 1D^19,20^.

A major question remains: Can the simulations be made efficient, so that universality is achieved in the strong sense? As mentioned earlier, the constructions used by ref. ^17^ to prove Theorem CMP18 are only efficient if the target Hamiltonian have the same or lower spatial dimensionality as the simulator Hamiltonian. When attempting to simulate a 3D target Hamiltonian (or worse, all-to-all interacting, such as the SYK model^25,26^) by a 2D simulator, however, the simulation requires exponentially large interaction energy *J*_*ij*_ = 2^Ω(poly(*n*))^. Similar exponential overhead is required in refs. ^18–20^ for simulating local Hamiltonians with general connectivity.

### Strongly universal Hamiltonian simulators

With the definitions and background introduced, we now show how to use the families of 2D Hamiltonians proposed in ref. ^17^ to achieve not only *weak universality* but also *strong universality* for analog simulation.

#### Theorem 1

Any ${{\mathcal{S}}}$-Hamiltonian family on the 2D square lattice is strongly universal, as long as ${{\mathcal{S}}}$ is non-2SLD. In particular, it’s sufficient for ${{\mathcal{S}}}$ to contain only a single interaction (such as Heisenberg or XY-interaction), implying that there are semi-translation-invariant Hamiltonians in 2D that are strongly universal.

We further show that strong universality can even be achieved by 1D Hamiltonians with nearest-neighbor interactions. However, we give up translation-invariance in the process.

#### Theorem 2

There is a strongly universal family of 1D Hamiltonians consisting of nearest-neighbor interactions acting on a line of 8-dimensional particles.

The interactions used in Theorem 2 arise from the circuit-to-Hamiltonian construction applied to a quantum circuit in 1D. These interactions are tailored to represent the gates that make up a general 1D circuit, and thus, they do not possess any translation-invariance.

To prove our results, we note that previous constructions^17–20^ incurred an exponential overhead because they relied purely on perturbative gadgets to reduce the connectivity of qudits. Specifically, in these constructions, in order to reduce degree in the interaction graph from Ω(*n*) to *O*(1) so that the Hamiltonian can be embedded on a finite-dimensional lattice, it is necessary to apply $\Omega (\log n)$ rounds of perturbative gadgets, each of which roughly halves the degree. Since the required interaction energy increases polynomially for each application of the perturbation gadget (i.e., *J*⟼[*J*poly(*n*)]^*c*^ for some constant *c* > 1), the final Hamiltonian requires interaction energy scaling as ${J}_{{\mathrm{fi}}nal}={n}^{{c}^{\Omega (\log n)}}={2}^{\Omega ({{\mbox{poly}}}(n))}$ in these constructions.

To circumvent this problem, we start from an idea from ref. ^27^ (which was recently applied in ref. ^20^) in which a transformation is performed by first mapping the target Hamiltonian *H* to a quantum circuit (implementing a phase estimation algorithm with respect to *e*^−*iHt*^) and only then mapping back to another Hamiltonian ${H}^{{\prime} }$ using a variant of the circuit-to-Hamiltonian construction^28^. The reason to use the intermediate quantum circuit step is that, unlike Hamiltonians, circuits can be straightforwardly made “sparse"—i.e., each qubit in the circuit is only acted upon by a few gates. This can be done by swapping qubits to fresh ancilla qubits after every computational gate. The sparsity of the circuit is what enables the final Hamiltonian to be embeddable in a 1D or 2D lattice.

Our construction of Hamiltonian simulators in 2D first utilizes techniques from earlier Hamiltonian complexity literature^3,29^. To obtain a semi-translation-invariant simulator, we borrow additional gadgets from ref. ^17^. To obtain a 1D Hamiltonian simulator, we employ a construction of QMA-complete 1D Hamiltonians from refs. ^30,31^ to simulate the quantum circuit using nearest-neighbor Hamiltonian interactions on a line of 8-dimensional particles. Although naïve application of these methods creates issues such as nonlocality of the encoding in the simulation, we are able to address them by carefully modifying the constructions. See the Methods section for more details on the proofs.

While our discussion thus far is restricted to simulating target Hamiltonians that are *O*(1)-local, the universal families we have constructed can actually simulate more complicated Hamiltonians.

#### Definition 6

(digitally simulable): An *n*-qudit Hamiltonian *H* is *digitally simulable* if for any *ϵ* > 0 and *t* one can construct a quantum circuit *U* consisting of poly(*n*, *t*, *ϵ*^−1^) one- or two-qubit gates (drawn from some universal gate set) such that ∥*U* − *e*^−*iHt*^∥≤*ϵ*, and the description of *U* is computable in poly(*n*, *t*, *ϵ*^−1^) time.

Since the aforementioned efficient simulators are based on circuit-to-Hamiltonian constructions, they easily generalize to any digitally simulable Hamiltonian:

#### Theorem 3

Any digitally simulable Hamiltonian has an efficient analog simulation by a Hamiltonian from any of the strongly universal families described in Theorems 1 and 2.

### Analog vs. dynamics simulation

The results above refer to analog Hamiltonian simulation as defined in Definition 2. Nevertheless, there is an alternative, commonly adopted notion of Hamiltonian simulation which focuses only on simulating time-evolution dynamics. While at first the distinction may appear negligible, there are in fact important differences between the two notions. We define dynamics simulation, borrowing inspiration from Definition 2, as:

#### Definition 7

(dynamics simulation): A Hamiltonian ${H}^{{\prime} }$ is an (*η*, *ϵ*)-*dynamics simulation* of an *n*-qudit Hamiltonian *H* if for any time *t* ≥ 0, there exist (possibly time-dependent) encoding maps ${{{\mathcal{E}}}}_{in}^{state}(\rho,t)$ and ${{{\mathcal{E}}}}_{out}^{state}(\rho,t)$, which are *η*-local state-encodings as in Definition 1, such that for any density matrix *ρ*_0_, we have $\parallel {e}^{-i{H}^{{\prime} }{t}^{{\prime} }}{{{\mathcal{E}}}}_{in}^{state}({\rho }_{0},t){e}^{i{H}^{{\prime} }{t}^{{\prime} }}-{{{\mathcal{E}}}}_{out}^{state}({e}^{-iHt}{\rho }_{0}{e}^{iHt},t){\parallel }_{1}\le \epsilon t$, where ${t}^{{\prime} }={{\mbox{poly}}}(t,n)$.

See ref. ^32^ for a related definition, which allows ${{{\mathcal{E}}}}_{state}^{in}$ and ${{{\mathcal{E}}}}_{state}^{out}$ to be any polynomial-sized quantum circuit.

As we shall see, dynamics simulation is a somewhat weaker notion than analog simulation. We first show that an analog simulator is automatically a dynamics simulator; a somewhat restricted version of the converse statement also holds:

#### Claim 1

If ${H}^{{\prime} }$ is a (Δ, *η*, *ϵ*)-analog simulation of *H* as in Definition 2, then ${H}^{{\prime} }$ is an (*η*, 2*ϵ*)-dynamics simulation of *H*. Conversely, if ${H}^{{\prime} }$ is an (*η*, 0)-dynamics simulation of *H* with time-independent encodings ${{{\mathcal{E}}}}_{in}^{state}(\rho,t)={{{\mathcal{E}}}}_{out}^{state}(\rho,t)={{{\mathcal{E}}}}^{state}(\rho )$ and ${t}^{{\prime} }=t$, then ${H}^{{\prime} }+cI$ restricted to a certain subspace is a (Δ, *η*, 0)-analog simulation of *H*, for some offset *c* and some Δ≥∥*H*∥.

See Supplementary Note 1 for a detailed proof.

**Fig. 1:**
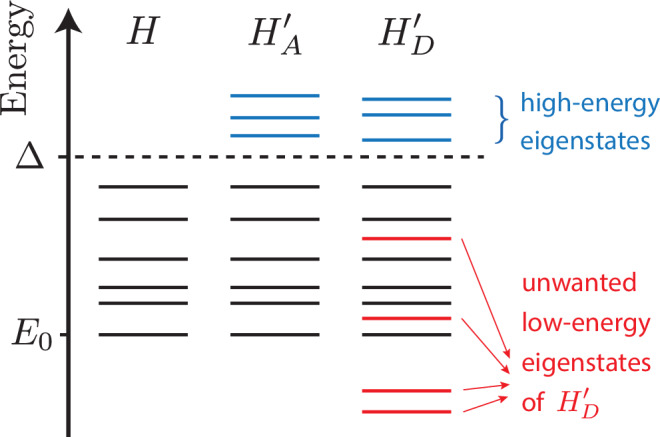
Sketch of the spectra of a Hamiltonian ${H},$ its analog simulator ${H}^{\prime}_{A},$ and its dynamics simulator ${H}^{\prime}_{D}$. The black eigenstates of ${H}^{\prime}_{A}\,{{\rm{and}}}\,{H}^{\prime}_{D}$ are those in the range of $\epsilon^{state}(1)$ and encode the eigenstates of ${{H}}$. In contrast to the analog simulator ${{H}}_{{A}}$ however, the dynamics simulator ${{H}}_{{D}}$ may also contain other eigenstates, such as the unwanted low-energy eigenstates (red). Leakage into these states, possibly driven by thermal fluctuations, can cause ${{H}}_{{D}}$ to fail to be a good analog simulator. Nevertheless, if the leakage can be suppressed, then ${{H}}_{{D}}$ restricted to the range of $\epsilon^{state}(1)$ (black) and the high-energy eigenstates (blue) may still serve as an analog simulation of ${H}$.

A dynamics simulator need not function as an analog simulator of the full Hamiltonian because it can possess unwanted low-energy eigenstates without counterparts in the target system. An example is illustrated in Fig. 1. Such a dynamics simulator only becomes an analog simulator after restriction to the desired eigenstates; however, any uncontrolled leakage into the unwanted eigenstates could cause the analog simulation to fail. In particular, thermal fluctuations and other noise sources can drive the dynamics simulator to populate these low-energy eigenstates, preventing it from faithfully reproducing the equilibrium thermodynamical properties of the target Hamiltonian. Moreover, a dynamics simulator may allow a nonlinear time parametrization ${t}^{{\prime} }=f(t)$ that can distort the spectrum significantly and further obstruct faithful analog simulation.

Thus, dynamics simulation is strictly less powerful than analog simulation. It is interesting to explore the notion of universality in the context of dynamics simulation. We define weak and strong universality for dynamics simulator by slightly modifying Definition 3 in the obvious way. Then Claim 1 implies that the families of Hamiltonians in Theorems 1 and 2, which are strongly universal for analog simulations, are also strongly universal for dynamics simulation.

#### Corollary 1

Any ${{\mathcal{S}}}$-Hamiltonian family on the 2D square lattice is strongly universal for dynamics simulation of any Hamiltonian, as long as ${{\mathcal{S}}}$ is non-2SLD. When ${{\mathcal{S}}}$ contains only a single interaction, we have semi-translation-invariant families of Hamiltonians in 2D that are strongly universal for dynamics simulation. There is also a family of 1D Hamiltonians for 8-dimensional particles that is strongly universal for dynamics simulation (but with no semi-translation-invariance).

We remark that strong universality for dynamics simulation is easier to achieve than for analog simulation. For example, an alternative proof of strong universality of 2D dynamics simulator, can also be shown from previous results regarding universal quantum computation using quantum walk Hamiltonians ^33^. Furthermore, ref. ^32^ has constructed a family of 1D, fully translation-invariant Hamiltonians based on quantum cellular automata on 14,580-dimensional particles that is strongly universal for dynamics simulation, albeit with a non-local encoding. Nevertheless, these Hamiltonians are not analog simulators due to the presence of exponentially many low-energy states corresponding to other computations that can be encoded in the initial state of the automaton. Hence, such simulators likely exhibit less noise-resilience. We do not know how to generalize ref. ^32^’s construction to achieve strongly universal analog simulators with full translation-invariance.

## Discussion

In this work, we have significantly improved the prospects of universal analog quantum simulation by showing that efficient universal simulation is possible with simple families of Hamiltonians embedded in low-dimensional space. Unlike the exponential overheads generally required in previous works^17–20^, our results show that only polynomial overheads in both particle number and interaction energy are sufficient to simulate any digitally simulable Hamiltonian with arbitrary connectivity by some universal Hamiltonians embedded in 1D or 2D. We note that the polynomial overhead in the interaction energy cannot be brought down to a constant using finite-dimensional Hamiltonian simulators, by an earlier impossibility result^27^. We remark that even though there are weakly universal, fully translation-invariant families of Hamiltonians in 1D and 2D^19,20^, the interaction energy in those Hamiltonians scales exponentially with the size of the target Hamiltonian.

We have also clarified the difference between analog Hamiltonian simulation and dynamics simulation. Although often referred to interchangeably, we show that analog simulation is a strictly more powerful notion than dynamics simulation, which only aims to reproduce the time-evolution under the Hamiltonian. Strongly universal dynamics simulators are also easier to construct than their analog counterparts. For example, we can show that 1D Hamiltonians with full translation-invariance are strongly universal for dynamics simulation, but not yet for analog simulation.

An independent concurrent work^34^ proved that a family of Hamiltonians is strongly (or in their terminology, efficiently) universal if and only if it is closed under addition and QMA-complete under what they call faithful reductions. They showed that the circuit-to-Hamiltonian construction used in ref. ^28^ to prove QMA-completeness of general local Hamiltonians (without restriction on geometry) can be modified to satisfy these conditions. Their proof strategy for showing faithfulness bears some similarities to our approach (e.g., idling trick). To check that their condition holds for the Hamiltonian families considered in the current paper, one would need a new proof that these 2D and 1D Hamiltonians are QMA-complete under faithful reduction; we believe that this can be done by adopting arguments in our proof of Proposition 2 and Theorem 2. This would provide a somewhat different proof of the strong universality of the Hamiltonian families in Theorems 1 and 2.

Several natural open questions remain for future work. Are there strongly universal semi-translation-invariant analog Hamiltonian simulators in 1D? The known gadgets to simulate general interactions with a single type of interaction seem to require ancilla particles placed in more than 1D; hence, new gadgetry appears necessary. Are there strongly universal Hamiltonians with full translation-invariance in any constant dimensions? Such constructions would be very helpful for near-term implementations. We note this is impossible when the only free parameter of the Hamiltonians is the number of particle ${n}^{{\prime} }$; since such Hamiltonians can be described by $O(\log {n}^{{\prime} })$ bits of information, they cannot represent general Hamiltonians on *n* qudits that are described by poly(*n*) bits unless ${n}^{{\prime} }=\exp ({{\mbox{poly}}}(n))$. Indeed, refs. ^19,20^ constructed such families of Hamiltonians that are weakly universal, but cannot be strongly universal; this also implies that not all weakly universal families are strongly universal.

To obtain full-TI strongly universal analog simulators, we may consider relaxing translation-invariance by allowing more free parameters in the interaction operator. This allows us to encode general target Hamiltonians using the poly(*n*) bits describing the interaction. Ref. ^20^ has done this to maintain *O*(poly(*n*)) particles overhead in the simulator, but their construction still requires exponential overhead in the interaction energy. Nevertheless, it is likely possible to efficiently simulate any full-TI Hamiltonian by a family of full-TI Hamiltonians, where the description of both Hamiltonians is $O(\log n)$ bits. It would be worthwhile to investigate whether these constructions can be improved, or otherwise prove impossibility of strong universality of full-TI Hamiltonians.

While this work constructs efficient universal analog quantum simulators using simple 1D or 2D systems, with only polynomial overheads in all resources, the constructions presented here are not optimized for actual implementation, and there is much room for improvement for practical applications. The main contributor to the resource overhead in our analysis is the non-perturbative Hamiltonian-circuit-Hamiltonian pipeline, which is currently dominated by the Trotter decomposition of *e*^−*iHt*^ in the phase-estimation subroutine and amplified by the energy penalties involved in the circuit-Hamiltonian construction. These overheads could potentially be improved by replacing Trotterization with QSVT-based^35^ method which has an exponentially better dependence on the precision parameter. Furthermore, we can consider a hybrid approach that applies the costly non-pertubative mapping to only the high-degree or high-locality terms in the input Hamiltonian while handling the rest with cheaper perturbative gadgets. On the hardware side, superconducting qubit platforms^16^ with native or programmable Heisenberg/XY couplings are well-suited to implement our 2D universal Hamiltonians; it would also be interesting to construct other universal families of Hamiltonians tailored to neutral atom and trapped ion platforms^36,37^ whose geometries are more flexible. More generally, there are useful trade-offs between structural constraints and resource costs; for example, relaxing semi-translation-invariance can reduce resource overheads by dispensing with gadgets that enforce a single interaction type. We further anticipate that leveraging other methods, such as the recently proposed dissipative gadgets^38^, may achieve simulation with better resource scaling. As experimental realizations of analog quantum simulators develop rapidly, we hope our work helps to expand their scope to simulate all physical systems and tackle classically intractable problems.

## Methods

Here, we sketch the construction of universal Hamiltonian simulators for proving Theorem 1 and Theorem 2.

### Overview

The starting point of our proofs for both Theorem 1 and 2 is as follows. We note that previous constructions^17–19,29^ relied purely on perturbative gadgets to reduce the connectivity of qudits. As mentioned earlier in the Results section, reducing the degree in the interaction graph from Ω(*n*) to *O*(1) required $\Omega (\log n)$ rounds of perturbative gadget, yielding a final Hamiltonian with interaction energy blowing up as 2^Ω(poly(*n*))^.

Instead, we reduce the connectivity of the qudits in our construction by first mapping the target Hamiltonian *H* to a quantum circuit performing the phase estimation algorithm with respect to *e*^−*iHt*^. Using standard techniques, including Trotter decomposition^39^, we can embed this circuit in 1D, using only nearest-neighbor gates on a line of qubits, while still applying the desired phase estimation with sufficient accuracy. For any given energy eigenstate $| {\psi }_{E}\rangle$, where $H| {\psi }_{E}\rangle=E| {\psi }_{E}\rangle$, this circuit writes down the energy eigenvalue as a bit-string in some ancilla qubits to approximately yield $| {\psi }_{E}\rangle \otimes | E\rangle$. This is the first step, summarized in Proposition 1.

After the first step (Proposition 1), in which the target Hamiltonian is replaced by a phase estimation circuit operating in 1D, we wish to map the resulting circuit back to a Hamiltonian that can be embedded in a 1D line or 2D lattice.

In the 2D case, we do this by applying ideas from refs. ^3,29^ to convert the 1D circuit given in Proposition 1 to a 2D spatially sparse Hamiltonian. Roughly speaking, spatial sparsity means that each qubit participates in only a constant number of local interactions or gates, arranged in a spatially local way on the 2D plane (see Definition 8). Note that a circuit that uses only nearest-neighbor gates in 1D is not necessarily spatially sparse since each qubit can participate in *O*(poly(*n*)) gates. Nevertheless, we can utilize swap gates to transform the 1D circuit into a 2D spatially sparse circuit non-perturbatively, where the second spatial dimension plays the role of time in the circuit. Having enforced the 2D spatial sparsity of the circuit, we next apply the Feynman-Kitaev circuit-to-Hamiltonian construction^28^ to obtain a 2D spatially sparse Hamiltonian *H*_circuit_ with roughly the same connectivity as the circuit. Importantly, this Hamiltonian has an exponentially large degeneracy of ground states: for each eigenstate of *H*, we have a groundstate of *H*_circuit_ corresponding to the computational history of the phase estimation circuit running on that eigenstate as input.

In order to restore the spectral features of *H* in *H*_circuit_, we apply the additional trick of imposing bit-wise energy penalties on the energy bit-string ancilla qubits to match the energy of the eigenstate. The states corresponding to the different computational histories of different eigenstates of *H* exhibit unwanted entanglement between the original qubits and the ancilla qubits, which causes decoherence in the simulation. For example, the state $| \psi \rangle={\sum }_{E}| {\psi }_{E}\rangle$ in the original system is roughly mapped to $| \widetilde{\psi }\rangle \approx {\sum }_{E}| {\psi }_{E}\rangle \otimes | E\rangle$. This decoherence can be repaired by the tricks of “uncomputing” the unwanted entanglement with the ancilla, and “idling” at the end of the (virtual) circuit, so that $| \psi \rangle$ is instead mapped to $| {\psi }^{{\prime} }\rangle \approx ({\sum }_{E}| {\psi }_{E}\rangle )\otimes | a\rangle$ where $| a\rangle$ is some explicit state on the ancilla qubits.

This part of the construction, obtaining *H*_circuit_, is summarized in Proposition 2.

We then prove strong universality of 2D Hamiltonians by converting *H*_circuit_, which is spatially sparse in 2D, to a semi-translation-invariant Hamiltonian ${H}^{{\prime} }$ on a 2D square lattice using gadgets from ref. ^17^. It can be shown that all steps of the construction incur only polynomial overhead in the interaction energy and the number of qubits, even if we restrict to 2D lattice Hamiltonians with Heisenberg or XY-type interactions. Hence, such families of semi-TI 2D Hamiltonians are strongly universal, concluding our proof of Theorem 1.

**Fig. 2:**
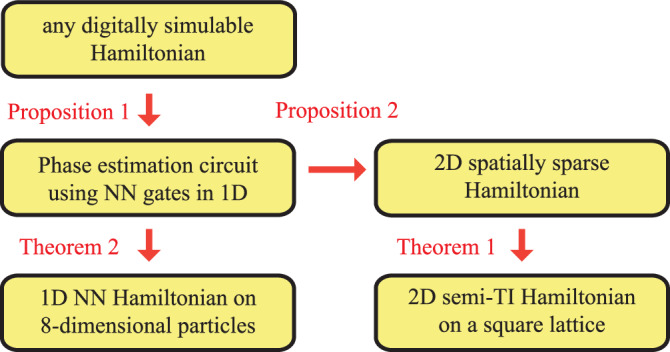
Overview of our constructions of 1D and 2D universal families of Hamiltonians. NN = nearest-neighbor. TI = translation-invariant.

When attempting to obtain a universal family of Hamiltonians in 1D, we cannot directly apply the above methods to the 1D circuit to obtain a 1D Hamiltonian. Specifically, there are two difficulties: (1) embedding both the space and time dimension of the 1D circuit in 1D, and (2) enforcing semi-translation-invariance in 1D. The proofs of the 1D and 2D cases thus diverge after the first step of Proposition 1. Figure 2 explains the proof structure for both 2D and 1D cases.

Instead, we construct a family of 1D Hamiltonians that simulate 1D phase estimation circuits by combining the tools of uncomputing and idling with previous circuit-to-Hamiltonian constructions that yielded QMA-complete 1D Hamiltonians^30,31^ to achieve our Theorem 2. The interactions in this Hamiltonian family are nearest-neighbor operators that enforce a set of transition rules, so that the zero-energy eigenstates are those corresponding to performing the circuit computation correctly. This address the first difficulty. However, we are currently unable to enforce semi-translation-invariance, so the second difficulty is left open. This is because the interactions here vary according to the gate sequence in the 1D circuit, and the gadgets from ref. ^17^ to obtain semi-TI only works in 2D. After applying the 1D circuit-to-Hamiltonian construction from refs. ^30,31^, we recover the spectral properties of *H* by imposing bit-wise energy penalties and repair decoherence via uncomputing and idling, similar to the 2D case. We note a naïve application of their construction produces an encoding that is not local in the sense of Definition 1. Nevertheless, we are able to restore the locality of encoding via a simple modification to the transition rules.

In both constructions of the 1D and 2D families, our non-perturbative techniques avoid the exponential blow-up in the required interaction energy, which brings the scaling of the energy overhead *J*_final_ down to only *O*(poly(*n*)).

Theorem 3 follows since both our 2D and 1D constructions are efficient for any input Hamiltonian *H* as long as the phase estimation circuit on *e*^*iHt*^ resulting from Proposition 1 is polynomial in size. If *H* is digitally simulable, then this is true by Definition 6.

In the remainder of the “Methods” section, we provide more detailed sketches of the steps in our constructions.

### The 1D phase estimation circuit (proposition 1)

We first show—and this is fairly standard—that one can construct a phase estimation circuit ${U}_{{\rm{PE}}}^{{\rm{NN}}}$ that would take eigenstates of *H* as input and (approximately) write down their energy on ancilla qubits, using only nearest-neighbor gates acting on a line of qubits.

Given any *n*-qubit digitally simulable Hamiltonian *H*, let us write the decomposition in its energy eigenbasis as $H={\sum }_{\mu }{E}_{\mu }| {\psi }_{\mu }\rangle \langle {\psi }_{\mu }|$, where $0\le {E}_{\mu }\le {E}_{\max }$ without loss of generality. Note the upper bound ${E}_{\max }=O({{\mbox{poly}}}(n))$ can be computed without knowledge of the energy eigenvalues, e.g., by adding up the spectral norm of individual local terms of *H*.

Ideally, we want a phase estimation circuit ${U}_{PE}^{ideal}$ that acts on any input state of the form $| \psi \rangle | {0}^{m}\rangle={\sum }_{\mu }{c}_{\mu }| {\psi }_{\mu }\rangle | {0}^{m}\rangle$ in the following way

3 $${U}_{{\rm{PE}}}^{{\rm{ideal}}}{\sum }_{\mu }{c}_{\mu }| {\psi }_{\mu }\rangle | {0}^{m}\rangle={\sum }_{\mu }{c}_{\mu }| {\psi }_{\mu }\rangle | {E}_{\mu }\rangle,$$

where $| {E}_{\mu }\rangle=| {\varphi }_{\mu,1}{\varphi }_{\mu,2}\ldots \rangle$, and *φ*_*μ*_ = 0. *φ*_*μ*,1_*φ*_*μ*,2_…  is the binary representation of the real number ${\varphi }_{\mu }={E}_{\mu }/{E}_{\max }$.

In reality, however, there are various approximations we must make to construct the phase estimation circuit in 1D. First, we cannot get infinite precision in the estimation of *E*_*μ*_, nor can we get an efficient approximation to within exponential precision (polynomially many bits). Hence, we restrict the first $s=O(\log (n))$ qubits of the *m*-qubit ancilla register to encode the energy *E*_*μ*_ as $| {\widetilde{E}}_{\mu }\rangle=| {\varphi }_{1}{\varphi }_{2}\ldots {\varphi }_{s}\rangle \otimes | {rest}_{\mu }\rangle$. Here $| {rest}_{\mu }\rangle$ is some arbitrary state in the remaining *m* − *s* ancilla qubits.

**Fig. 3:**
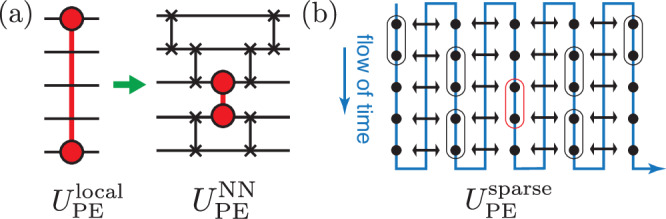
Illustration of converting any circuit to a spatially sparse one. **a** Adding swap gates to make a 2-qubit gate (red) acting on distant qubits nearest-neighbor. **b** Adding new qubits so that the execution of gates is in a spatially local sequence.

Second, we want to implement such an approximation of ${U}_{PE}^{ideal}$, where $| {E}_{\mu }\rangle$ is replaced with $| {\widetilde{E}}_{\mu }\rangle$, using only a polynomial number of local gates. Note that the phase estimation is applied to the unitary *e*^*iHt*^, which is not a local unitary operation. Nonetheless, for *O*(1)-local Hamiltonians, we can easily apply the standard Trotter decomposition^39^ to split *e*^*iHt*^ into a product of local gates. We also want to use a discrete set of 2-qubit universal gates, which can be done by invoking the Solovay-Kitaev theorem^22^. This gives us ${U}_{PE}^{local}$, which will have some error $\zeta=\parallel ({U}_{{\rm{PE}}}^{{\rm{local}}}-{U}_{{\rm{PE}}}^{{\rm{ideal}}})| \psi \rangle | {0}^{m}\rangle \parallel$. This error *ζ* can be made small using only *O*(poly(*n*, *ζ*^−1^)) resources. Furthermore, this fact is true by definition for any digitally simulable Hamiltonian. Then, as shown in Fig. 3(a), we make all gates to be nearest-neighbor on a 1D line by adding swap gates, obtaining ${U}_{{\rm{PE}}}^{{\rm{NN}}}$. This fact is summarized as Proposition 1, which we prove more formally in Supplementary Note 2:

#### Proposition 1

Given any *n*-qubit digitally simulable Hamiltonian $H={\sum }_{\mu }{E}_{\mu }| {\psi }_{\mu }\rangle \langle {\psi }_{\mu }|$, with a promise that the energies *E*_*μ*_’s lie in the interval $[0,{E}_{\max }]$, one can construct a circuit ${U}_{{\rm{PE}}}^{{\rm{NN}}}$ consisting of poly(*n*, *ζ*^−1^) gates acting on *n* + *m* qubits arranged on a 1D line with 1- or 2-qubit nearest-neighbor gates chosen from a discrete universal gate set, where *m* = poly(*n*), such that for any normalized input state ${\sum }_{\mu }{c}_{\mu }| {\psi }_{\mu }\rangle$

4 $$\left|\left| {U}_{{\rm{PE}}}^{{\rm{NN}}}{\sum }_{\mu }{c}_{\mu }| {\psi }_{\mu }\rangle | {0}^{m}\rangle -{\sum }_{\mu }{c}_{\mu }| {\psi }_{\mu }\rangle | {\widetilde{E}}_{\mu }\rangle \right|\right| \le \zeta$$

where the first *s* < *m* qubits of $| {\widetilde{E}}_{\mu }\rangle$ can be read off as an *s*-bit approximation of the real number ${\varphi }_{\mu }={E}_{\mu }/{E}_{\max }$.

### Constructing 2D semi-TI strongly universal Hamiltonians

To prove Theorem 1, we want to simulate any local (or digitally simulable) Hamiltonian *H* with an ${{\mathcal{S}}}$-Hamiltonian on a 2D square lattice with polynomial overhead, for any non-2SLD ${{\mathcal{S}}}$. Based on the 1D phase estimation circuit produced by Proposition 1, we construct a spatially sparse circuit Hamiltonian *H*_circuit_ that simulates our original target Hamiltonian *H* (Proposition 2). Then the (semi-translation-invariant) ${{\mathcal{S}}}$-Hamiltonian simulator on the 2D lattice is obtained from *H*_circuit_ by applying a series of gadgets. The full technical proof in given in Supplementary Note 3, but we will sketch the essential ideas below. To that end, we borrow the notion of spatial sparsity from refs. ^17,29^ and generalize it to circuits:

#### Definition 8

(Spatial sparsity of Hamiltonians and circuits (adapted from ref. ^29^)): A Hamiltonian on *n*-qudits is *spatially sparse* if its interaction hypergraph is one where (i) every vertex participates in *O*(1) hyper-edges, and (*ii*) there is a straight-line drawing in the plane such that every hyper-edge overlaps with *O*(1) other hyper-edges, and the surface covered by every hyper-edge is *O*(1). Moreover, we say a quantum circuit $U={\prod }_{t=1}^{T}{U}_{t}$ is *spatially sparse* if there is a placement of the qudits in the two-dimensional plane such that (i) each qudit participates in *O*(1) gates, and (ii) the spatial supports of *U*_*t*_ and *U*_*t*+1_ are only *O*(1) distance apart for all *t*, each covering *O*(1) contiguous area.

With the definition of spatial sparsity at hand, we now formally state the Proposition we want to show:

#### Proposition 2

Given any *O*(1)-local *n*-qudit Hamiltonian *H* with ∥*H*∥ = *O*(poly(*n*)) and *d* = *O*(1), one can construct a spatially sparse 5-local Hamiltonian *H*_circuit_ that efficiently simulates *H* (c.f. Definition 2) to precision (Δ, *η*, *ϵ*), with Δ = *O*(*ϵ*^−1^∥*H*∥^2^ + *η*^−1^∥*H*∥). *H*_circuit_ has *O*(poly(*n*, *ϵ*^−1^)) terms and qubits, and interaction energy at most *O*(poly(*n*, *η*^−1^, *ϵ*^−1^)).

To prove this Proposition, we start from the 1D phase estimation circuit ${U}_{{\rm{PE}}}^{{\rm{NN}}}$ obtained using Proposition 1 and construct a spatially sparse circuit using swap gates. To do this, we first make ${U}_{{\rm{PE}}}^{{\rm{NN}}}$ into a spatially sparse version, ${U}_{{\rm{PE}}}^{{\rm{sparse}}}$, by moving it from the line to a 2D grid. As visualized in Fig. 3(b), starting from the leftmost column of qubits, we apply just one nearest-neighbor gate before swapping all qubits to the next column and applying the next gate, and so on, getting us to ${U}_{{\rm{PE}}}^{{\rm{sparse}}}$. By ordering the gates in a snake-like fashion similar to refs. ^3,29^, we make sure that each qubit participates in only a constant number of gates, and that the temporally proximate gates in the sequence have spatially proximate support.

Before converting the circuit back to a Hamiltonian, we need to address the issue of the entanglement between each energy eigenstate and the ancilla register that has the energy bit-string, as evident in Eq. (3). This incoherence between different eigenstates causes a large error for the simulation if not addressed. We repair this error by running the circuit backwards ("uncomputing”) and then adding *L* identity gates at the end ("idling”), so that most of the computational history of each eigenstate is simply the state itself. We then get a new circuit ${U}_{circuit}={({\mathbb{1}})}^{L}{({U}_{{\rm{PE}}}^{{\rm{sparse}}})}^{{\dagger} }{({\mathbb{1}})}^{s}{U}_{{\rm{PE}}}^{{\rm{sparse}}}$, which is spatially sparse as long as ${U}_{{\rm{PE}}}^{{\rm{sparse}}}$ is. Note we inserted *s* identity gates before applying ${({U}_{{\rm{PE}}}^{{\rm{sparse}}})}^{{\dagger} }$ so that we can examine the energy bit-string bit-by-bit before it is uncomputed. Although ${U}_{circuit}={\mathbb{1}}$ is ultimately a trivial operation, the circuit’s nontrivial temporal structure allows its computational history to encode the spectral information about the target Hamiltonian.

Subsequently, we apply Kitaev’s circuit-to-Hamiltonian mapping^28^ to convert the spatially sparse, “uncomputed" circuit *U*_circuit_ to a spatially sparse Hamiltonian *H*_circuit_. To do this in a spatially sparse way, we observe that in the circuit-to-Hamiltonian construction^28^, each clock qubit corresponds to a single gate; then in our construction, that clock qubit is placed geometrically in the middle of the hyper-edge connecting the qubits participating in the gate, interacting with only nearby qubits. Hence, the spatially sparsity of *H*_circuit_ is guaranteed by the spatial sparsity of *U*_circuit_. The energy of the eigenstates of *H* is restored by adding an appropriate bit-wise energy penalty on the *s* qubits where the energy is written, while we idle between ${U}_{{\rm{PE}}}^{{\rm{sparse}}}$ and ${({U}_{{\rm{PE}}}^{{\rm{sparse}}})}^{{\dagger} }$ [see Supplementary Equation (26)]. We then use perturbative arguments (such as those shown in ref. ^40^) to show that *H*_circuit_ simulates *H* with polynomially small error, with only polynomial overheads. This proves Proposition 2.

Finally, to prove Theorem 1, we map the spatially sparse Hamiltonian *H*_circuit_ to a Hamiltonian in a universal family on the 2D square lattice with additional polynomial overhead. This is done via a sequence of reductions, with known techniques^17,29^: We first convert *H*_circuit_ to a real-valued Hamiltonian by doubling the number of qubits, and encoding any Pauli *Y*’s into a pair of *Y* ⊗ *Y*. We then remove all Pauli *Y*’s, and reduce the locality of the Hamiltonian to 2-local via applications of perturbative gadgets. This is subsequently converted to a spatially sparse ${{{\mathcal{S}}}}_{0}$-Hamiltonian where ${{{\mathcal{S}}}}_{0}=\{XX+YY+ZZ\}$ or {*XX* + *YY*}. Throughout this sequence of reductions, the spatial sparsity of the Hamiltonian is preserved, and both the interaction energy and qubit number only increase polynomially as the original system size and the target precision parameters (Δ, *η*, *ϵ*). This spatially sparse ${{{\mathcal{S}}}}_{0}$-Hamiltonian involving only Pauli-interactions without *Y*’s can then be mapped to an ${{{\mathcal{S}}}}_{0}$-Hamiltonian on the 2D lattice. Since the input Hamiltonian is spatially sparse, this mapping only incurs polynomial overhead in the interaction energy as shown in refs. ^17,29^. Furthermore, ref. ^17^ has shown that ${{{\mathcal{S}}}}_{0}$-Hamiltonian on a 2D square lattice can be simulated by any ${{\mathcal{S}}}$-Hamiltonian on a 2D square lattice for any non-2SLD ${{\mathcal{S}}}$ (c.f. Definition 5), with polynomial overhead. We have thus provided a construction that allows any such family of ${{\mathcal{S}}}$-Hamiltonians on the 2D square lattice to efficiently simulate any local Hamiltonian with arbitrary geometry.

### Constructing 1D universal Hamiltonians

We now describe how to construct a strongly universal family of Hamiltonian simulators in 1D. To prove this result as stated in Theorem 2, we extend the circuit-to-1D-Hamiltonian constructions in refs. ^30,31^. These constructions were originally used to show QMA-completeness of Hamiltonians involving nearest-neighbor interaction on a 1D line of particles, by using them to encode the outcome of any circuit computation in their ground state energy.

The basic idea in these constructions is as follows. Suppose we are given any quantum circuit consisting of *R* rounds of nearest-neighbor gates on *n* qubits in 1D, such as ${U}_{{\rm{PE}}}^{NN}$, where each round is of the form *U*_*n*−1,*n*_ ⋯ *U*_23_*U*_12_ (some may be the identity gate). We consider an equivalent circuit on a line of 2*nR* particles, divided into *R* blocks, where each block encodes the computational state of the original *n* qubits. In this equivalent circuit, a single round of nearest-neighbor gates is applied in each block before the qubits are moved to the next block, where subsequent gates can be performed. The 8 internal states of each particle are necessary to store both the computational state of the original qubit and marker states that allow us to locally distinguish different stages of the computation of each particle (e.g., whether a gate has already been applied or needs to be applied).

We want to apply these constructions to simulate general Hamiltonians. Following the same idea as in the proof of Theorem 1, we first convert the target Hamiltonian *H* to a phase estimation circuit ${U}_{{\rm{PE}}}^{NN}$ using nearest-neighbor gates on a line of qubits as in Proposition 1. Then, we use the scheme in ref. ^31^ as well as our tricks of “uncomputing” and “idling” to map the circuit to a 1D Hamiltonian of nearest neighbor-interactions, and penalize the energy bit-string so that we simulate the full spectral properties of *H*. However, a naïve application of this scheme would yield a highly nonlocal encoding, since the idling part of the circuit corresponds to simply moving the computational qubits down the line, causing the encoded eigenstates of *H* to be delocalized over many blocks of particles. To circumvent this issue, we modified the scheme so that the idling step is done without moving the computational qubits, while maintaining the consistency of all transition rules so that the legal computational history states are spectrally gapped from the rest. Consequently, the eigenstates of *H* are encoded in the qubit-subspace of some 8-state qudits from just one block of the line, yielding a local encoding. See Supplementary Note 4 for the full proof.

## Supplementary information

Supplementary Information

Transparent Peer Review file

## Data Availability

No datasets were generated or analyzed during the current study.
