# Supplementary material for: Universal Hamiltonian simulators in one and two dimensions
Source: Nat Commun. 2026 Apr 21;17:6888. doi: 10.1038/s41467-026-71686-4 (PMC13388968; doi:10.1038/s41467-026-71686-4)
Supplement: Supplementary file 1 — Supplementary Information [file 41467_2026_71686_MOESM1_ESM.pdf]

Supplementary Information for "Universal Hamiltonian Simulators"

Leo Zhou[1, 2, 3, *] and Dorit Aharonov[4, †]

[1] *Electrical and Computer Engineering Department, University of California, Los Angeles, CA 90095, USA*
[2] *Walter Burke Institute for Theoretical Physics, Caltech, Pasadena, California 91125, USA*
[3] *Institute for Quantum Information and Matter, Caltech, Pasadena, California 91125, USA*
[4] *School of Computer Science and Engineering, The Hebrew University, Jerusalem 91904, Israel*

CONTENTS

I. Supplementary Note 1 – Relationship between analog and dynamics simulations 1

II. Supplementary Note 2 – A 1D nearest-neighbor implementation of phase estimation circuit 3

III. Supplementary Note 3 – Proof that spin models on 2D lattice are strongly universal 5
A. Efficient and spatially sparse Hamiltonian simulator 5
B. Proof of Theorem 1 – Strongly Universal Hamiltonian on 2D Square Lattice 10

IV. Supplementary Note 4 – Proof that 1D nearest-neighbor Hamiltonians are strongly universal 12
A. Preliminaries 12
B. Proof of Theorem 2 13

References 15

I. SUPPLEMENTARY NOTE 1 – RELATIONSHIP BETWEEN ANALOG AND DYNAMICS SIMULATIONS

In this section, we prove Claim 1 in the main text about the relationship of analog and dynamics simulation.

We start by restating the definitions of local encodings and Hamiltonian simulations given in the main text, which will also be used in the rest of the Supplementary Information.

Definition 1 (Local encoding, adapted from [1]). *Consider an encoding map* $\mathcal{E} : \mathrm{Herm}((\mathbb{C}^d)^{\otimes n}) \to \mathrm{Herm}((\mathbb{C}^{d'})^{\otimes n'})$ *that takes Hermitian operators on* n *qudits (*d*-dimensional particles) into operators acting on* n' d'*-dimensional particles (where* $d^n \le {d'}^{n'}$*). We say* $\mathcal{E}$ *is an* η-local encoding *if we can write*

$$\mathcal{E}(A) = V(A \otimes P + \bar{A} \otimes Q)V^\dagger, \tag{1}$$

such that V *is an isometry satisfying* $\|V - V_{\mathrm{loc}}\| \le \eta$ *for some local isometry* $V_{\mathrm{loc}} = \bigotimes_{i=1}^n V_i$*, where each* V_i *is an isometry acting on one qudit in the original system.* P *and* Q *are locally orthogonal projectors, i.e.,* P *and* Q *are orthogonal projectors and* $\forall i\ \exists$ *orthogonal projectors* P_i, Q_i *acting on the same subsystem as* V_i *such that* $P_i Q_i = 0$*,* $P_i P = P$ *and* $Q_i Q = Q$ *(see also [1, Supporting Information, Theorem 15]).* $\bar{A}$ *is the complex conjugation of* A*.* $\|\cdot\|$ *is the spectral norm.*

When $\eta = 0$*, we say* $\mathcal{E}$ *is a* local encoding.

Furthermore, $\mathcal{E}^{\mathrm{state}}$ *is an* η*-local state-encoding if it is of the form* $\mathcal{E}^{\mathrm{state}}(\rho) = \mathcal{E}'(\rho)/\operatorname{Tr}(\mathcal{E}'(\rho))$ *for some* η*-local encoding* $\mathcal{E}'$*.*

Definition 2 (Analog Hamiltonian simulation, adapted from [1]). *A Hamiltonian* H' *is a* (Δ, η, ϵ)-simulation *of an* n*-qudit Hamiltonian* H *if there exists an* η*-local encoding* $\mathcal{E}_\eta$ *as in Definition 1 such that*

1. $\mathcal{E}_\eta(\mathbb{1}) = P_{\le \Delta(H')}$;

---

* leoxzhou@ucla.edu
† dorit.aharonov@gmail.com

2. $\|H'_{\leq\Delta} - \mathcal{E}_\eta(H)\| \leq \epsilon$.

Here, $P_{\leq\Delta(H')}$ is the projector onto the subspace of eigenstates of H' with eigenvalue $\leq \Delta$, and $H'_{\leq\Delta} = P_{\leq\Delta(H')}H'$ is the restriction of H' onto these states. We say the simulation is efficient *if both the number of particles in H' and its maximum energy $\|H'\|$ are at most $O(\mathrm{poly}(n, \eta^{-1}, \epsilon^{-1}, \Delta))$, and the description of H' is efficiently computable.*

Definition 3 (dynamics simulation)**.** *A Hamiltonian H' is an (η, ϵ)-*dynamics simulation *of an n-qudit Hamiltonian H if for any time $t \geq 0$, there exist (possibly time-dependent) encoding maps $\mathcal{E}^{\mathrm{state}}_{\mathrm{in}}(\rho, t)$ and $\mathcal{E}^{\mathrm{state}}_{\mathrm{out}}(\rho, t)$, which are η-local state-encodings as in Definition 1, such that for any density matrix ρ_0, we have*

$$\left\| e^{-iH't'} \mathcal{E}^{\mathrm{state}}_{\mathrm{in}}(\rho_0, t) e^{iH't'} - \mathcal{E}^{\mathrm{state}}_{\mathrm{out}}(e^{-iHt}\rho_0 e^{iHt}, t) \right\|_1 \leq \epsilon t, \tag{2}$$

where $t' = \mathrm{poly}(t, n)$.

We now restate Claim 1 from the main text, which we prove in the remainder of this section:

Claim 1. *If H' is a (Δ, η, ϵ)-analog simulation of H as in Definition 2, then H' is an $(\eta, 2\epsilon)$-dynamics simulation of H. Conversely, if H' is an $(\eta, 0)$-dynamics simulation of H with time-independent encodings $\mathcal{E}^{\mathrm{state}}_{\mathrm{in}}(\rho, t) = \mathcal{E}^{\mathrm{state}}_{\mathrm{out}}(\rho, t) = V\rho V^\dagger$ and $t' = t$, then $H' + cI$ restricted to a certain subspace is a $(\Delta, \eta, 0)$-analog simulation of H, for some offset c and some $\Delta \geq \|H\|$.*

Proof. First we show the easier direction: a good analog simulator is a good dynamics simulator. This follows somewhat straightforwardly from the properties of analog simulation (see also [1, Supporting Information, Corollary 29]). Given that H' is a (Δ, η, ϵ)-simulation of H, then by Definition 2 there is an η-local encoding $\mathcal{E}_\eta(A) = V(A \otimes P + \bar{A} \otimes Q)V^\dagger$ such that $\mathcal{E}_\eta(\mathbb{1}) = P_{\leq\Delta(H')}$ and $\|H'_{\leq\Delta} - \mathcal{E}_\eta(H)\| \leq \epsilon$. Observe that

$$e^{-i\mathcal{E}_\eta(H)t} = V \exp[-i(H \otimes P + \bar{H} \otimes Q)t]V^\dagger = V(e^{-iHt} \otimes P + e^{-i\bar{H}t} \otimes Q)V^\dagger, \tag{3}$$

where the last equality follows from the fact that P and Q are orthogonal projectors and $PQ = 0$.

Suppose for now $\mathrm{rank}(P) \geq 1$. Let σ be any state where $P\sigma = \sigma$. Let $\mathcal{E}^{\mathrm{state}}(\rho) = V(\rho \otimes \sigma)V^\dagger$, which is an η-local state-encoding since $\mathcal{E}_\eta$ is an η-local encoding. Then

$$\mathcal{E}^{\mathrm{state}}(e^{-iHt}\rho_0 e^{iHt}) = V(e^{-iHt}\rho_0 e^{iHt} \otimes \sigma)V^\dagger = e^{-i\mathcal{E}_\eta(H)t}\mathcal{E}^{\mathrm{state}}(\rho_0)e^{i\mathcal{E}_\eta(H)t}, \tag{4}$$

where the last equality uses the fact that $Q\sigma = 0$.

Applying these identities, we have

$$\begin{aligned} \left\| e^{-iH't}\mathcal{E}^{\mathrm{state}}(\rho_0)e^{iH't} - \mathcal{E}^{\mathrm{state}}(e^{-iHt}\rho_0 e^{iHt}) \right\|_1 &= \left\| e^{-iH't}\mathcal{E}^{\mathrm{state}}(\rho_0)e^{iH't} - e^{-i\mathcal{E}_\eta(H)t}\mathcal{E}^{\mathrm{state}}(\rho_0)e^{i\mathcal{E}_\eta(H)t} \right\|_1 \\ &= \left\| e^{-iH'_{\leq\Delta}t}\mathcal{E}^{\mathrm{state}}(\rho_0)e^{iH'_{\leq\Delta}t} - e^{-i\mathcal{E}_\eta(H)t}\mathcal{E}^{\mathrm{state}}(\rho_0)e^{i\mathcal{E}_\eta(H)t} \right\|_1 \\ &\leq 2\left\| e^{-iH'_{\leq\Delta}t} - e^{-i\mathcal{E}_\eta(H)t} \right\| \leq 2\left\| H'_{\leq\Delta} - \mathcal{E}_\eta(H) \right\| t \leq 2\epsilon t. \end{aligned} \tag{5}$$

The second line uses the fact that $\mathcal{E}^{\mathrm{state}}(\rho_0)$ is in the subspace $P_{\leq\Delta(H')}$. In the last line, the first inequality comes from the fact that $\|ACA^\dagger - BCB^\dagger\|_1 \leq (\|A\| + \|B\|)\|A - B\|\|C\|_1$ (see [1, Supporting Information, Lemma 17]). The second inequality follows from the fact that $\partial_t[e^{iAt}e^{-iBt} - \mathbb{1}] = e^{iAt}(A - B)e^{-iBt}$. Letting $\mathcal{E}^{\mathrm{state}}_{\mathrm{in}}(\cdot, t) = \mathcal{E}^{\mathrm{state}}_{\mathrm{out}}(\cdot, t) = \mathcal{E}^{\mathrm{state}}(\cdot)$ and $t' = t$, this shows that H' is an $(\eta, 2\epsilon)$-dynamics simulation of H as in Definition 3.

For the other case of $\mathrm{rank}(P) = 0$, we necessarily have $\mathrm{rank}(Q) \geq 1$. Let σ be any state where $Q\sigma = \sigma$, and $\mathcal{E}^{\mathrm{state}}(\rho) = V(\bar{\rho} \otimes \sigma)V^\dagger$. Then $\mathcal{E}^{\mathrm{state}}(e^{-iHt}\rho_0 e^{iHt}) = e^{i\mathcal{E}_\eta(H)t}\mathcal{E}^{\mathrm{state}}(\rho_0)e^{-i\mathcal{E}_\eta(H)t}$, which is a time-reversed simulation. Hence, letting $t' = -t$, the same argument as above shows that H' is an $(\eta, 2\epsilon)$-dynamics simulation of H.

Now we show that the converse direction is almost true. Given that H' is an $(\eta, 0)$-dynamics simulation of H with time-independent encodings $\mathcal{E}^{\mathrm{state}}_{\mathrm{in}}(\rho, t) = \mathcal{E}^{\mathrm{state}}_{\mathrm{out}}(\rho, t) = V\rho V^\dagger$ and $t' = t$. Then Definition 3 implies that for any input state ρ_0 and time $t \geq 0$,

$$e^{-iH't}V\rho_0 V^\dagger e^{iH't} = Ve^{-iHt}\rho_0 e^{iHt}V^\dagger. \tag{6}$$

Let us write $H = \sum_\mu E_\mu |\psi_\mu\rangle\langle\psi_\mu|$ in its eigendecomposition. Consider $\rho_0 = |\psi_\mu\rangle\langle\psi_\mu|$ as input state, then

$$e^{-iH't}V|\psi_\mu\rangle\langle\psi_\mu|V^\dagger e^{iH't} = V|\psi_\mu\rangle\langle\psi_\mu|V^\dagger, \tag{7}$$

which implies that $|\psi'_\mu\rangle := V|\psi_\mu\rangle$ is an eigenstate of H'. Let us denote E'_μ as the (currently unknown) corresponding eigenvalue. Next, for any two eigenstate $|\psi_1\rangle$ and $|\psi_2\rangle$ of H, consider $(|\psi_1\rangle + |\psi_2\rangle)/\sqrt{2}$ as input state. Then

$$Ve^{-iHt}\rho_0 e^{iHt}V^\dagger = \frac{1}{2}(|\psi'_1\rangle\langle\psi'_1| + |\psi'_2\rangle\langle\psi'_2| + e^{i\delta_{12}t}|\psi'_1\rangle\langle\psi'_2| + e^{-i\delta_{12}t}|\psi'_2\rangle\langle\psi'_1|), \tag{8}$$

where we have denoted $\delta_{12} = E_2 - E_1$. Let $\delta'_{12} = E'_2 - E'_1$, then

$$e^{-iH't}V\rho_0 V^\dagger e^{iH't} = \frac{1}{2}(|\psi'_1\rangle\langle\psi'_1| + |\psi'_2\rangle\langle\psi'_2| + e^{i\delta'_{12}t}|\psi'_1\rangle\langle\psi'_2| + e^{-i\delta'_{12}t}|\psi'_2\rangle\langle\psi'_1|). \tag{9}$$

Since these two are equal for any $t \geq 0$, we must have $\delta'_{12} = \delta_{12}$. Hence, H' preserves the relative difference between any two eigenvalues of H, but there could be an arbitrary offset between the two set of eigenvalues $\{E_\mu\}_\mu$ and $\{E'_\mu\}_\mu$. Let $E_0 = \min_\mu E_\mu$ and $E'_0 = \min_\mu E'_\mu$. Let $\mathcal{L} = \mathrm{span}\{|\psi'_\mu\rangle\}_\mu$ be the subspace spanned by the encoded eigenstates of H, possibly augmented by excited states of H' with energy $\geq \max_\mu E'_\mu$. Then $H' + (E_0 - E'_0)\mathbb{1}$ restricted to $\mathcal{L}$ is a $(\Delta, \eta, 0)$-simulation of H for some $\Delta \geq \|H\|$, with η-local encoding $\mathcal{E}_\eta(A) = VAV^\dagger$. □

II. SUPPLEMENTARY NOTE 2 – A 1D NEAREST-NEIGHBOR IMPLEMENTATION OF PHASE ESTIMATION CIRCUIT

In this section, we formally show that given any digitally simulable Hamiltonian H (including all local Hamiltonians), one can construct a phase estimation circuit such that the energy of any input eigenstate of H can be written down as bits on some ancilla qubits to $O(\log n)$ bit precision with $O(1/\mathrm{poly}(n))$ error in the state. The proof uses standard techniques such as Trotter decomposition and Solovay-Kitaev algorithm. In particular, we show that this can be done with a circuit acting on a line of qubits with nearest-neighbor gates. This will serve as the first step of our efficient construction of universal Hamiltonian simulators.

Proposition 1 (formal)**.** *Consider any digitally simulable Hamiltonian $H = \sum_a H_a = \sum_\mu E_\mu|\psi_\mu\rangle\langle\psi_\mu|$ acting on n qubits, where we assume w.l.o.g. that $0 \leq E_\mu \leq E_{\max}$ for some known number $E_{\max} = O(\mathrm{poly}(n))$. For any $s = O(\log n)$ and $\zeta > 0$, we can construct a phase estimation circuit $U_{\mathrm{PE}}^{\mathrm{NN}}$ consisting of $O(\mathrm{poly}(n, \zeta^{-1}))$ 1- or 2-qubit nearest neighbor gates drawn from any universal gate set, acting on a line of $n + m$ qubits, where $m = O(\mathrm{poly}(n))$ such that the following is true: For any normalized state $\sum_\mu c_\mu |\psi_\mu\rangle$, the circuit $U_{\mathrm{PE}}^{\mathrm{NN}}$ satisfies*

$$\left\| U_{\mathrm{PE}}^{\mathrm{NN}} \sum_\mu c_\mu |\psi_\mu\rangle |0^m\rangle - \sum_\mu c_\mu |\psi_\mu\rangle |\tilde{E}_\mu\rangle |\mathrm{rest}_\mu\rangle \right\| \leq \zeta \tag{10}$$

where $|\tilde{E}_\mu\rangle = |\varphi_{\mu,1}\varphi_{\mu,2}\varphi_{\mu,3}\cdots\varphi_{\mu,s}\rangle$ is the s-bit truncated representation of $\varphi_\mu = E_\mu/E_{\max} = 0.\varphi_{\mu,1}\varphi_{\mu,2}\varphi_{\mu,3}\cdots$ with $\varphi_{\mu,j} \in \{0,1\}$, and $|\mathrm{rest}_\mu\rangle$ is some unimportant, normalized state on the remaining $m - s$ ancilla qubits.

Proof. Let us first consider the standard implementation of quantum phase estimation algorithm circuit U_{PE}. Here, the circuit uses the evolution operator $u_j = e^{iH\tau 2^{j-1}}$ under H, where $\tau = 2\pi/E_{\max}$, and writes phase of the eigenvalues of $u_1 = e^{iH\tau}$ on some ancilla qubits. Note the eigenvalues of u_1 are $e^{i2\pi\varphi_\mu}$, where $\varphi_\mu = E_\mu\tau/(2\pi)$. Since $0 \leq \varphi_\mu \leq 1$, we can write $\varphi_\mu = 0.\varphi_{\mu,1}\varphi_{\mu,2}\varphi_{\mu,3}\ldots$, where $\varphi_{\mu,j} \in \{0,1\}$ as its binary representation.

Ideally, the action of the phase estimation circuit on input states $\{|\psi_\mu\rangle|0^m\rangle\}_{\mu=1}^{2^n}$ is

$$U_{\mathrm{PE}}^{\mathrm{ideal}} |\psi_\mu\rangle |0^m\rangle = |\psi_\mu\rangle |E_\mu\rangle, \tag{11}$$

where $|E_\mu\rangle = |\varphi_{\mu,1}\rangle \otimes |\varphi_{\mu,2}\rangle \otimes \cdots \otimes |\varphi_{\mu,m}\rangle$ gives a m-bit string that exactly represents the real number $E_\mu/E_{\max}$.

In reality, there are two sources of errors that cause our desired 1D implementation of the phase estimation circuit to deviate from $U_{\mathrm{PE}}^{\mathrm{ideal}}$.

Error 1: finite bit-precision.— The first error is due to the fact that the energy eigenvalues don't generally have finite-bit-precision representation. Let us denote $\tilde{E}_\mu = 2\pi\tilde{\varphi}_\mu/\tau$ as an approximate value of the energy E_μ with s-bit precision, where $\tilde{\varphi}_\mu = 0.\varphi_{\mu,1}\varphi_{\mu,2}\varphi_{\mu,3}\cdots\varphi_{\mu,s}$.

The error comes from the fact that generally, $|E_\mu - \tilde{E}_\mu| = O(2^{-s})$ is non-zero. In other words, since $\varphi_\mu \neq \tilde{\varphi}_\mu$, there's error from imprecise phase estimation. Let us consider a phase estimation circuit U_{PE} implemented to p-bit precision, where $p > s$. Let b_μ be the integer in the range $[0, 2^p - 1]$ such that $0 \leq \varphi_\mu - b_\mu/2^p \leq 2^{-p}$. It is well-known [2] that the action of the standard implementation of U_{PE} on any input state $|\psi_\mu\rangle|0\rangle$ result in the following state

$$U_{\mathrm{PE}} |\psi_\mu\rangle |0^m\rangle = |\psi_\mu\rangle \otimes \left(\frac{1}{2^p} \sum_{k,\ell=0}^{2^p-1} e^{-i2\pi k\ell/2^p} e^{i2\pi\varphi_\mu k} |\ell\rangle \right) \otimes |\mathrm{extra}_\mu\rangle = |\psi_\mu\rangle \otimes \left(\sum_{\ell=0}^{2^p-1} \alpha_\ell^\mu |\ell\rangle \right) \otimes |\mathrm{extra}_\mu\rangle \tag{12}$$

where $|\ell\rangle = |\ell_1 \cdots \ell_p\rangle$ is the binary representation of ℓ,

$$\alpha_\ell^\mu = \frac{1}{2^p}\sum_{k=0}^{2^p-1}\left[e^{i2\pi(\varphi_\mu-\ell/2^p)}\right]^k = \frac{1}{2^p}\left[\frac{1-e^{i2\pi(2^s\varphi_\mu-\ell)}}{1-e^{i2\pi(\varphi_\mu-\ell/2^p)}}\right], \tag{13}$$

and $|\mathrm{extra}_\mu\rangle$ is some normalized state on the remaining $m-p$ ancilla qubits that may be storing some unimportant information about the computation upon input $|\psi_\mu\rangle|0^m\rangle$. The analysis from Sec. 5.2.1 in Ref. [2] shows that the probability of getting a state that is a distance of e integer away is

$$p_\mu^{\mathrm{error}}(e) \equiv \sum_{|\ell-b_\mu|>e} |\alpha_\ell^\mu|^2 \le \frac{1}{2(e-1)} \tag{14}$$

Note that we only care about the first $s<p$ bits, so we can choose $e = 2^{p-s}-1$. Hence,

$$\begin{aligned} U_{\mathrm{PE}}|\psi_\mu\rangle|0^m\rangle &= |\psi_\mu\rangle \otimes \left[\sum_{|\ell-b_\mu|\le e}\alpha_\ell^\mu|\ell\rangle + \sum_{|\ell-b_\mu|>e}\alpha_\ell^\mu|\ell\rangle\right]\otimes|\mathrm{extra}_\mu\rangle \\ &= |\psi_\mu\rangle\otimes\left(\sqrt{1-p_\mu^{\mathrm{error}}}\,|\tilde{E}_\mu\rangle|\mathrm{rest}_\mu^1\rangle + \sqrt{p_\mu^{\mathrm{error}}}\,|\mathrm{rest}_\mu^2\rangle\right)\otimes|\mathrm{extra}_\mu\rangle\,. \end{aligned} \tag{15}$$

Here, $|\tilde{E}_\mu\rangle = |\varphi_{\mu,1}\varphi_{\mu,2}\varphi_{\mu,3}\cdots\varphi_{\mu,s}\rangle$ represents the first s bits of φ_μ, $|\mathrm{rest}_\mu^1\rangle$ is a normalized superposition over the remaining $p-s$ qubit coming from the bitstrings which agree with $\varphi_\mu \approx b_\mu/2^p$ on the first s bits, and $|\mathrm{rest}_\mu^2\rangle$ is a normalized superposition of p-bit strings whose values deviate from the good estimate b_μ of φ_μ by more than e. Comparing this with the idealized output in Eq. (11), we can identify $|\mathrm{rest}_\mu\rangle := |\mathrm{rest}_\mu^1\rangle\otimes|\mathrm{extra}_\mu\rangle$, and observe that

$$(U_{\mathrm{PE}} - U_{\mathrm{PE}}^{\mathrm{ideal}})|\psi_\mu\rangle|0^m\rangle = |\psi_\mu\rangle|\mathrm{error}_\mu\rangle\,, \quad \text{where} \quad \||\mathrm{error}_\mu\rangle\|^2 \le 2p_\mu^{\mathrm{error}} = O(2^{-(p-s)}) \tag{16}$$

Thus, for any normalized state $|\psi\rangle = \sum_\mu c_\mu|\psi_\mu\rangle|0^m\rangle$, we have

$$\|(U_{\mathrm{PE}} - U_{\mathrm{PE}}^{\mathrm{ideal}})\sum_\mu c_\mu|\psi_\mu\rangle|0^m\rangle\|^2 = O(2^{-(p-s)}) \le \zeta/2 \tag{17}$$

where we choose, for example, $p = 2s + O(\log\zeta^{-1})$, and thus make this first source of error due to finite bit-precision to be smaller than $\zeta/2$ for any given $\zeta > 0$.

Error 2: local gate approximation of $e^{-iH\tau_j}$.— The second source of error is due to the fact that we need to implement the circuit U_{PE} using only 1 or 2-qubit gates, in order to ensure the corresponding circuit-Hamiltonian is local, The only non-local gates in the p-bit precise phase estimation algorithm that we need to address are the controlled-application of Hamiltonian evolution, $|0\rangle\langle 0|\otimes\mathbb{1} + |1\rangle\langle 1|\otimes u_j$, where $u_j = e^{-iH\tau_j}$, $\tau_j = 2^{j-1}\tau = 2^j\pi/E_{\max}$ and $j = 1, 2, \ldots, p$.

We address this by invoking the fact that H is digitally simulable, which by definition enables one to construct a quantum circuit W with $\mathrm{poly}(n,\tau,\xi^{-1})$ one- or two-qubit gates drawn from a universal set such that $\|W - e^{-iH\tau}\| \le \xi$. Choosing $\xi = \zeta/(4p)$ and applying this construction to $\{u_j\}_{j=1}^p$, this yield a phase-estimation circuit containing $\mathrm{poly}(n,\zeta^{-1})$ local gates acting on at most three qubits each. Further reduction to one- and two-qubit universal gates can be performed by using the Solovay-Kitaev algorithm [3], incurring $\mathrm{poly}\log\zeta$ overhead. The final circuit is $U_{\mathrm{PE}}^{\mathrm{local}}$ consisting of only $R_{\mathrm{local}} = \mathrm{poly}(n,\zeta^{-1})$ one- or two-qubit gates, satisfying

$$\|U_{\mathrm{PE}}^{\mathrm{local}} - U_{\mathrm{PE}}\| \le \zeta/2. \tag{18}$$

Lastly, we ensure that this circuit $U_{\mathrm{PE}}^{\mathrm{local}}$ only consists of nearest-neighbor gates by the following procedure:

1. Place all n qubits on a line with any pre-determined ordering.

2. Iterate over each gate U_t, $t = 1, \ldots R_{\mathrm{local}}$. If U_t acts on qubits that are not neighbors on the line, add a sequence S_t of swap gates on nearest neighbors in the circuit before U_t so that U_t acts on neighbors. Then add the same swap gates in reversed order in the circuit after U_t so that the qubits returned to their original order on the line. At the end of this step, the new circuit is of the form $U_{\mathrm{PE}}^{\mathrm{NN}} = \prod_{t=1}^{R_0}(S_t^\dagger U_t S_t)$, with $R_0 = O(R_{\mathrm{local}}N)$ gates, each only acting on a neighbors group of qubits. See Fig. 3(a) in the main text for an example.

Note $U_{\mathrm{PE}}^{\mathrm{NN}}$ is effectively equivalent to $U_{\mathrm{PE}}^{\mathrm{local}}$ since we are simply swapping qubits around.

Putting everything together, we have $U_{\mathrm{PE}}^{\mathrm{NN}} \equiv U_{\mathrm{PE}}^{\mathrm{local}} \approx U_{\mathrm{PE}} \approx U_{\mathrm{PE}}^{\mathrm{ideal}}$, where each of the approximate equality has error bounded by $\zeta/2$. In conclusion, for any $\zeta > 0$, we can construct a phase estimation circuit $U_{\mathrm{PE}}^{\mathrm{NN}}$ comprised of only $O(\mathrm{poly}(n, \zeta^{-1}))$ 1-qubit or 2-qubit nearest-neighbor gates on a line, such that its action is ζ-close to $U_{\mathrm{PE}}^{\mathrm{ideal}}$ on any normalized input state $|\psi\rangle = \sum_\mu c_\mu |\psi_\mu\rangle |0^m\rangle$:

$$\|(U_{\mathrm{PE}}^{\mathrm{NN}} - U_{\mathrm{PE}}^{\mathrm{ideal}}) |\psi\rangle |0^m\rangle \| \leq \zeta. \tag{19}$$

□

III. SUPPLEMENTARY NOTE 3 – PROOF THAT SPIN MODELS ON 2D LATTICE ARE STRONGLY UNIVERSAL

In this section, we show the details of the construction for strongly universal Hamiltonians on a 2D square lattice, proving the following theorem:

Theorem 1. *Any $\mathcal{S}$-Hamiltonian family on the 2D square lattice is strongly universal, as long as $\mathcal{S}$ is non-2SLD. In particular, it's sufficient for $\mathcal{S}$ to contain only a single interaction (such as Heisenberg or XY-interaction), implying that there are semi-translation-invariant Hamiltonians in 2D that are strongly universal.*

A. Efficient and spatially sparse Hamiltonian simulator

Before proving the strong universality of 2D spin-lattice models, we first prove the following result, where we show that any local Hamiltonian can be simulated by a spatially sparse Hamiltonian.

Proposition 2. *Given any $O(1)$-local n-qudit Hamiltonian H with $\|H\| = O(\mathrm{poly}(n))$ and $d = O(1)$, one can construct a spatially sparse 5-local Hamiltonian H_{circuit} that efficiently simulates H to precision (Δ, η, ϵ), with $\Delta = O(\epsilon^{-1}\|H\|^2 + \eta^{-1}\|H\|)$. H_{circuit} has $O(\mathrm{poly}(n, \epsilon^{-1}))$ terms and qubits, and interaction energy at most $O(\mathrm{poly}(n, \eta^{-1}, \epsilon^{-1}))$.*

To prove the above Proposition, we first prove two smaller results in Lemmas 1 and 2 about different aspects of using the Feynman-Kitaev circuit-to-Hamiltonian construction [4] for analog Hamiltonian simulation. The following concept of history states will be useful in our discussion:

Definition 4 (history state)**.** *Let $U = U_T \cdots U_2 U_1$ be a quantum circuit acting on $n + m$ qudits. Then for any input state $|\psi_\mu\rangle \in \mathbb{C}^{d^n}$ and $m = \mathrm{poly}(n)$, the* history state *with respect to U and $|\psi_\mu\rangle$ is the following*

$$|\eta_\mu\rangle = \frac{1}{\sqrt{T+1}} \sum_{t=0}^{T} \left(U_t \cdots U_2 U_1 |\psi_\mu\rangle |0^m\rangle^{\mathrm{anc}} \right) |1^t 0^{T-t}\rangle^{\mathrm{clock}} \tag{20}$$

We now state the first of the two lemmas, which describes a circuit-to-Hamiltonian transformation that can be used for analog Hamiltonian simulation, assuming an appropriate energy penalty Hamiltonian H_{out} can be constructed.

Lemma 1 (Circuit-Hamiltonian simulation)**.** *Let H be any target n-qudit Hamiltonian, with an orthonormal basis for eigenstates given by $\{|\psi_\mu\rangle\}_{\mu=1}^{d^n}$. Let $U = \prod_{t=1}^{T} U_t$ be a quantum circuit with T gates, each at most k-local. Let $\mathcal{L} = \mathrm{span}\{|\eta_\mu\rangle\}_{\mu=1}^{d^n}$ be the subspace of history states with respect to U and $\{|\psi_\mu\rangle\}$. Suppose there exists a Hamiltonian H_{out} such that*

$$\|VHV^\dagger - H_{out}|_{\mathcal{L}}\| \leq \epsilon/2 \tag{21}$$

where $\mathcal{E}(H) = VHV^\dagger$ is a local encoding, and $O|_{\mathcal{L}}$ means operator O restricted to subspace $\mathcal{L}$. Then for any $\eta > 0$, we can construct a Hamiltonian H_{circuit} from the description of U and H_{out} such that H_{circuit} is a (Δ, η, ϵ)-simulation of H with local encoding $\mathcal{E}$, where $\Delta \geq O(\epsilon^{-1}\|H_{out}\|^2 + \eta^{-1}\|H_{out}\|)$, as in Definition 2. The constructed H_{circuit} is $(k+3)$-local, has $O(T)$ terms and particles, and uses $O(\mathrm{poly}(n, T, \Delta))$ interaction energy. Furthermore, H_{circuit} is spatially sparse if the circuit U is spatially sparse.

The second lemma concerns an "idling" trick, where we transform a circuit so that its corresponding history states are modified to increase the simulation precision under a desired local encoding $\mathcal{E}(H) = H \otimes |\alpha\rangle\langle\alpha|$ (i.e. V is the isometry acting as $V|\psi\rangle = |\psi\rangle|\alpha\rangle$).

Lemma 2 (Idling to enhance simulation precision)**.** *Consider an uncomputed quantum circuit $U_D \cdots U_1 = \mathbb{1}$. Suppose we add L identity gates to the end of the circuit, so that we obtain a new circuit $U = \mathbb{1}^L U_D \cdots U_1$ with length $T = D+L$. Let $H = \sum_\mu E_\mu |\psi_\mu\rangle\langle\psi_\mu|$ be any Hamiltonian. Furthermore, let $|\eta_\mu\rangle$ be the history state with respect to U and $|\psi_\mu\rangle$, and suppose $H_{\rm eff} = \sum_\mu E_\mu |\eta_\mu\rangle\langle\eta_\mu|$. For any $\epsilon > 0$, if we choose $L = O(\frac{D\|H\|^2}{\epsilon^2})$, then there is an ancilla state $|\alpha\rangle$ such that $\|H \otimes |\alpha\rangle\langle\alpha| - H_{\rm eff}\| \leq \epsilon$.*

With the two lemmas stated, we can now prove our main Proposition 2:

Proof of Proposition 2. Given any $O(1)$-local n-qu$d$it Hamiltonian where d is a constant, we can easily convert it to an $O(1)$-local $O(n)$-qubit Hamiltonian by simply encoding each qu$d$it in the subspace of a group of $\lceil \log_2 d \rceil$ qubits. We can separate the extra states in this redundant encoding (when d is not a power of 2) from the relevant part of spectrum by adding to the Hamiltonian a local energy penalty term on acting each group with $\|H\| = O(\text{poly}(n))$ magnitude. Hence, we will call H the $O(1)$-local n-qubit Hamiltonian containing $O(\text{poly}(n))$-strength interactions obtained after this conversion.

Let us denote the normalized eigenstates of H as $|\psi_\mu\rangle$, with corresponding eigenvalues E_μ. We assume they are ordered such that $E_1 \leq E_2 \leq E_3 \leq \cdots \leq E_{2^n}$.

From Proposition 1, for any $s = O(\log n)$, we can construct a ζ-approximate, s-bit precise phase estimation circuit $U_{\rm PE}^{\rm NN}$ such that it acts on a line of $N = O(\text{poly}(n))$ qubits with $R_0 = O(\text{poly}(n, \zeta^{-1}))$ nearest-neighbor gates. We want to replace it with a spatially sparse circuit $U_{\rm PE}^{\rm sparse}$ with $O(R_0 N)$ qubits and gates (see Definition 8 in the main text). This can be done with polynomial overhead in the same way as done in Ref. [5, 6]. We begin by placing the N original qubits on the first column of a $N \times R_0$ grid of qubits. For column $i = 1, 2, \ldots, R_0$, we execute only the i-th gate from the circuit $U_{\rm PE}^{\rm NN}$, and other uninvolved qubits are acted on by identity gates. After each column, we swap the state of the qubit of column i to $i+1$. We order the execution of all the gates such that the gates from $U_{\rm PE}^{\rm NN}$ and identity gates are executed top-to-bottom, and the swap gates between column are executed from bottom-to-up [see Fig. 3(b) in the main text]. It is thus easy to see that in this new circuit $U_{\rm PE}^{\rm sparse}$, each qubit participates in at most 3 gates (up to two swap gates and a non-trivial gate), and the gate are executed in a spatially local sequence. Note the action of $U_{\rm PE}^{\rm sparse}$ is equivalent to $U_{\rm PE}^{\rm NN}$ up to re-ordering of the qubits, since we've only added swap gates. Thus, Proposition 1 gives us

$$\left\| U_{\rm PE}^{\rm sparse} \sum_\mu c_\mu |\psi_\mu\rangle |0^m\rangle - \sum_\mu c_\mu |\psi_\mu\rangle |\tilde{E}_\mu\rangle \right\| \leq \zeta \tag{22}$$

where $|\tilde{E}_\mu\rangle = |\varphi_{\mu,1}\varphi_{\mu,2}\varphi_{\mu,3}\ldots\varphi_{\mu,s}\rangle \otimes |\text{rest}_\mu\rangle$ contains the s-bit truncated representation of $\varphi_\mu = E_\mu/E_{\max} = 0.\varphi_{\mu,1}\varphi_{\mu,2}\varphi_{\mu,3}\cdots$, and $E_{\max}$ is the upper bound on the maximum energy of the target Hamiltonian H used in the construction of $U_{\rm PE}^{\rm NN}$. Let

$$\tilde{E}_\mu = E_{\max} \times (0.\varphi_{\mu,1}\varphi_{\mu,2}\varphi_{\mu,3}\ldots\varphi_{\mu,s}) = E_\mu + O(E_{\max} 2^{-s}) \tag{23}$$

be the truncated-approximation to the energy eigenvalue E_μ.

The new spatially sparse circuit $U_{\rm PE}^{\rm sparse}$ now has $t_0 = O(R_0 N) = O(\text{poly}(n, \zeta^{-1}))$ gates. However, the computational qubits are moved to places far from their original location due to the swap gates, and the history states are far from a local encoding of the original state. To address this issue, we construct the following uncomputed circuit with idling

$$U_{\rm circuit} = (\mathbb{1})^L U_{\rm PE}^{\rm sparse\dagger} (\mathbb{1})^s U_{\rm PE}^{\rm sparse}, \tag{24}$$

which we will soon transform into our spatially sparse Hamiltonian. Note this circuit is automatically spatially sparse since it's a composition of 4 spatially sparse circuits, and the computational states are conveniently moved back to the first column after the uncomputation. With the addition of $U_{\rm PE}^{\rm sparse\dagger}$ for uncomputing and $s+L$ idling identity gates, the entire circuit gate count is now $T = 2t_0 + s + L$. The s identity gates in the middle are used for local "measurements" of energy to s-bit precision by energy penalty Hamiltonian terms. The later $L = O((2t_0 + s)\|H\|^2/\epsilon^2) = O(\text{poly}(n, \zeta^{-1})/\epsilon^2)$ identity gates are used to ensure $O(\epsilon)$ simulation precision by a trivial encoding as in Lemma 2. The history states with respect to $U_{\rm circuit}$ and the eigenstates $\{|\psi_\mu\rangle\}$ of H are

$$|\eta_\mu\rangle = \frac{1}{\sqrt{T+1}} \sum_{t=0}^{T} \Big(U_t \cdots U_2 U_1 |\psi_\mu\rangle |0^m\rangle \Big) |1^t 0^{T-t}\rangle . \tag{25}$$

We now convert the circuit to a Hamiltonian $H_{\rm circuit}$ using the method described in Lemma 1, where H_{out} is chosen to be

$$H_{out} = (T+1) E_{\max} \sum_{b=1}^{s} 2^{-b} |1\rangle\langle 1|_b^{\rm anc} \otimes P^{\rm clock}(t_0 + b). \tag{26}$$

We also denote $P^{\text{clock}}(t) = |110\rangle\langle 110|^{\text{clock}}_{t-1,t,t+1}$, which projects onto legal clock states corresponding to time step t.

To show that H_{circuit} simulates the original Hamiltonian H, we first show that H_{out} restricted to the subspace of history states $\mathcal{L} = \text{span}\{|\eta_\mu\rangle : 1 \leq \mu \leq 2^n\}$ can be approximated by the following effective Hamiltonian

$$H_{\text{eff}} = \sum_\mu E_\mu |\eta_\mu\rangle\langle\eta_\mu|. \tag{27}$$

Consider arbitrary states $|\eta\rangle \in \mathcal{L}$. We write $|\eta\rangle = \sum_\mu a_\mu |\eta_\mu\rangle$, and observe

$$\langle\eta|H_{out}|\eta\rangle = E_{\max} \sum_{b=1}^{s} 2^{-b} \left[\sum_\nu a_\nu^* \langle\psi_\nu| \langle 0^m|\right] U_{\text{PE}}^{\text{sparse}\dagger} |1\rangle\langle 1|_b U_{\text{PE}}^{\text{sparse}} \left[\sum_\mu a_\mu |\psi_\mu\rangle |0^m\rangle\right] \tag{28}$$

Then using (22), we have

$$\langle\eta|H_{out}|\eta\rangle = E_{\max} \sum_{b=1}^{s} 2^{-b} \left[\sum_\nu a_\nu^* \langle\psi_\nu| \langle\tilde{E}_\nu| + \langle\zeta|\right] |1\rangle\langle 1|_b \left[\sum_\mu a_\mu |\psi_\mu\rangle |\tilde{E}_\mu\rangle + |\zeta\rangle\right] \tag{29}$$

where $|\zeta\rangle$ is some residual state vector with $\| \, |\zeta\rangle \, \| \leq \zeta$. Hence

$$|\langle\eta|H_{out} - H_{\text{eff}}|\eta\rangle| \leq \sum_\mu |a_\mu|^2 |\tilde{E}_\mu - E_\mu| + 2s\zeta E_{\max} \leq \max_\mu |\tilde{E}_\mu - E_\mu| + 2s\zeta E_{\max} \leq (2^{-s} + 2s\zeta) E_{\max} \tag{30}$$

We can ensure this is always less than $\epsilon/4$ by choosing for example

$$s = \log_2(8E_{\max}/\epsilon) = O(\log n + \log \epsilon^{-1}) \quad \text{and} \quad \zeta = \epsilon/(16 s E_{\max}) = O(1/\text{poly}(n, \epsilon^{-1})). \tag{31}$$

Hence,

$$|\langle\eta|H_{out} - H_{\text{eff}}|\eta\rangle| \leq \epsilon/4 \quad \forall |\eta\rangle \in \mathcal{L} \quad \implies \quad \|H_{\text{eff}} - H_{out}|_{\mathcal{L}}\| \leq \epsilon/4 \tag{32}$$

Furthermore, since we have added L idling gates such that $\|H \otimes |\alpha\rangle\langle\alpha| - H_{\text{eff}}\| \leq \epsilon/4$ for some ancilla state $|\alpha\rangle$ by Lemma 2, then together with (32) we have

$$\|H \otimes |\alpha\rangle\langle\alpha| - H_{out}|_{\mathcal{L}}\| \leq \epsilon/2. \tag{33}$$

Observe that we can rewrite $H \otimes |\alpha\rangle\langle\alpha| = VHV^\dagger$, where $V|\psi\rangle = |\psi\rangle|\alpha\rangle \; \forall |\psi\rangle \in \mathbb{C}^{2^n}$ is an isometry. Hence, by Lemma 1, for any $\eta > 0$, the constructed H_{circuit} simulates H to precision (Δ, η, ϵ), where $\Delta = O(\epsilon^{-1}\|H_{out}\|^2 + \eta^{-1}\|H_{out}\|) = O(\epsilon^{-1}\|H\|^2 + \eta^{-1}\|H\|)$. Note that H_{circuit} is spatially sparse since $U_{\text{PE}}^{\text{sparse}}$ is spatially sparse. Since $U_{\text{PE}}^{\text{sparse}}$ contains at most 2-local gates, which means H_{circuit} is at most 5-local. Furthermore, H_{circuit} contains $O(T) = O(\text{poly}(n, \zeta^{-1})/\epsilon^2) = O(\text{poly}(n, \epsilon^{-1}))$ terms (and qubits), with $O(\text{poly}(n, T, \epsilon^{-1}, \eta^{-1}, \|H_{out}\|)) = O(\text{poly}(n, \eta^{-1}, \epsilon^{-1}))$ interaction energy.

□

To finish the proof, we just need to prove Lemma 1 and 2. We start with the proof of Lemma 1.

Proof of Lemma 1. For a given circuit $U = U_T \cdots U_2 U_1$, the corresponding circuit-Hamiltonian is

$$H_{\text{circuit}} = H_0 + H_{out} \tag{34}$$

$$\text{where} \quad H_0 = J_{clock} H_{clock} + J_{prop} H_{prop} + J_{in} H_{in} \tag{35}$$

The role of H_0 is to isolate $\mathcal{L} = \text{span}\{|\eta_\mu\rangle\}$ as its zero-energy groundspace separated by a large spectral gap 2Δ from the rest of the eigenstates. Then H_{out} is used recover the eigenvalue structrue of H in the subspace $\mathcal{L}$, allowing H_{circuit} to simulate H.

Now we give the explicit form of the circuit-Hamiltonian. The first part of H_0 is

$$H_{clock} = \sum_{t=1}^{T-1} |01\rangle\langle 01|^{\text{clock}}_{t,t+1}, \tag{36}$$

which sets the legal state configurations in the clock register to be of the form $|t\rangle^{\text{clock}} \equiv |1^t 0^{T-t}\rangle^{\text{clock}}$. Then, we simulate the state propagation under the circuit using

$$\begin{aligned} H_{prop} &= \sum_{t=1}^{T} H_{prop,t}, \qquad (37)\\ \text{where} \quad H_{prop,t} &= \mathbb{1} \otimes |100\rangle\langle 100|^{\text{clock}}_{t-1,t,t+1} - U_t \otimes |110\rangle\langle 100|^{\text{clock}}_{t-1,t,t+1} \\ &\quad - U_t^\dagger \otimes |100\rangle\langle 110|^{\text{clock}}_{t-1,t,t+1} + \mathbb{1} \otimes |110\rangle\langle 110|^{\text{clock}}_{t-1,t,t+1} \quad \text{for} \quad 1 < t < T, \\ H_{prop,1} &= \mathbb{1} \otimes |00\rangle\langle 00|^{\text{clock}}_{12} - U_1 \otimes |10\rangle\langle 00|^{\text{clock}}_{12} - U_1^\dagger \otimes |00\rangle\langle 10|^{\text{clock}}_{12} + \mathbb{1} \otimes |10\rangle\langle 10|^{\text{clock}}_{12}, \\ \text{and} \quad H_{prop,T} &= \mathbb{1} \otimes |10\rangle\langle 10|^{\text{clock}}_{T-1,T} - U_T \otimes |11\rangle\langle 10|^{\text{clock}}_{T-1,T} - U_T^\dagger \otimes |10\rangle\langle 11|^{\text{clock}}_{T-1,T} + \mathbb{1} \otimes |11\rangle\langle 11|^{\text{clock}}_{T-1,T}. \end{aligned}$$

These terms check the propagation of states from time $t-1$ to t is correct. Now, we also need to ensure that the input states are valid, i.e. ancilla qudits are in the state $|0^m\rangle^{\text{anc}}$ when $t = 0$ (i.e., the clock register is $|0^T\rangle^{\text{clock}}$). This can be done using

$$H_{in} = \sum_{i=1}^{m} (\mathbb{1} - |0\rangle\langle 0|)_i^{\text{anc}} \otimes |0\rangle\langle 0|^{\text{clock}}_{t_{\min}(i)}, \qquad (38)$$

$$\text{where} \quad t_{\min}(i) = \min\{t : U_t \text{ acts nontrivially on ancilla qudit } i\}.$$

In other words, for each ancilla qudit i, H_{in} penalizes the ancilla if it's not in the state $|0\rangle$ before it is first used by the $t_{\min}(i)$-th gate. Note that H_{circuit} has $O(T)$ terms, each of which is most $(k+3)$-local when U_t are k-local. If U is spatially sparse, then it is easy to see that H_{circuit} is also spatially sparse.

Note that $H_0 \mathcal{L} = 0$. We then need to lower bound the spectral gap of H_0, i.e. $\lambda_1(H_0|_{\mathcal{L}^\perp})$, where $\lambda_1(H)$ denotes the lowest eigenvalue of H. To that end, let us denote the following subspaces:

$$\mathcal{S}_{clock} = \text{span}\{|\psi\rangle\,|y\rangle\,|1^t 0^{T-t}\rangle : |\psi\rangle \in \mathbb{C}^{d^n} \text{ and } |y\rangle \in \mathbb{C}^{d^m}, 0 \le t \le T\}, \qquad (39)$$

$$\mathcal{S}_{prop} = \text{span}\{|\eta_\mu, y\rangle \equiv \frac{1}{\sqrt{T+1}} \sum_{t=0}^{T} \Big(U_t \cdots U_2 U_1 |\psi\rangle\,|y\rangle \Big)\,|1^t 0^{T-t}\rangle : 1 \le \mu \le d^n, 0 \le y \le d^{n-1}\}. \qquad (40)$$

Note that $\mathcal{L} \subset \mathcal{S}_{prop} \subset \mathcal{S}_{clock}$. Let us denote $\tilde{A} = \mathcal{A} \cap \mathcal{L}^\perp$ for any subspace $\mathcal{A}$. Note $H_{clock}\mathcal{S}_{clock} = 0$, $H_{prop}\mathcal{S}_{prop} = 0$, $H_{in}\mathcal{L} = 0$. We will use the following Projection Lemma 3:

Lemma 3 (Projection Lemma, adapted from [7])**.** *Let $H = H_1 + H_2$ be the sum of two Hamiltonians operating on some Hilbert space $\mathcal{S}_0 = \mathcal{S} \oplus \mathcal{S}^\perp$. Assuming that H_2 has a zero-energy eigenspace $\mathcal{S} \subseteq \mathcal{S}_0$ so that $H_2\mathcal{S} = 0$, and that the minimum eigenvalue $\lambda_1(H_2|_{\mathcal{S}^\perp}) \ge J > 2\|H_1\|$, then*

$$\lambda_1(H_1|_{\mathcal{S}}) - \frac{\|H_1\|^2}{J - 2\|H_1\|} \le \lambda_1(H) \le \lambda_1(H_1|_{\mathcal{S}}). \qquad (41)$$

In particular, if $J \ge K\|H_1\|^2 + 2\|H_1\| = O(K\|H_1\|^2)$, we have $\lambda_1(H_1|_{\mathcal{S}}) - \frac{1}{K} \le \lambda_1(H) \le \lambda_1(H_1|_{\mathcal{S}})$. ♦

Applying the above Lemma successively to H_0, we obtain

$$\begin{aligned} \lambda_1(H_0|_{\mathcal{L}^\perp}) &\ge \lambda_1\left[(J_{prop}H_{prop} + J_{in}H_{in})|_{\tilde{\mathcal{S}}_{clock}}\right] - \frac{1}{K} \quad \text{if} \quad J_{clock} = O(K\|J_{prop}H_{prop} + J_{in}H_{in}\|^2) \qquad (42)\\ &\ge \lambda_1\left[(J_{in}H_{in})|_{\tilde{\mathcal{S}}_{prop}}\right] - \frac{2}{K} \quad \text{if} \quad J_{prop}/T^2 = O(K\|J_{in}H_{in}\|^2) \qquad (43) \end{aligned}$$

where we used the fact that $\lambda_1(H_{clock}|_{\mathcal{S}^\perp_{clock}}) \ge 1$, and $\lambda_1(H_{prop}|_{\mathcal{S}^\perp_{prop}}) \ge c/T^2$ for some constant c. We now lower bound (43). Let us denote $\hat{n} = \mathbb{1} - |0\rangle\langle 0|$. Then within $\mathcal{S}_{clock}$, we can rewrite

$$H_{in}|_{\mathcal{S}_{clock}} = \sum_{i=1}^{m} \hat{n}_i^{\text{anc}} \otimes \sum_{0 \le t \le t_{\min}(i)} |t\rangle\langle t|^{\text{clock}} = \sum_{t=0}^{\max_i t_{\min}(i)} H_{in,t} \qquad (44)$$

$$\text{where} \quad H_{in,t} = \sum_{\{i:\ t \le t_{\min}(i)\}} \hat{n}_i^{\text{anc}} \otimes |t\rangle\langle t|^{\text{clock}}.$$

In particular, $H_{in,t=0} = \sum_{i=1}^m \hat{n}_i^{\text{anc}} \otimes |t=0\rangle\langle t=0|$. Thus, for any $|\eta_\mu, y\rangle, |\eta_\nu, y'\rangle \in \tilde{\mathcal{S}}_{prop}$, where necessarily $y, y' > 0$, we have

$$\begin{aligned} \langle \eta_\nu, y' | H_{in,t=0} | \eta_\mu, y\rangle &= \frac{1}{T+1} \langle \psi_\nu | \langle y' | H_{in,t=0} | \psi_\mu\rangle | y\rangle \\ &= \frac{1}{T+1} \delta_{\mu\nu} \langle y' | \sum_{i=1}^m \hat{n}_i^{\text{anc}} | y\rangle = \frac{1}{T+1} \delta_{\mu\nu} \delta_{y,y'} \times w(y), \end{aligned} \tag{45}$$

where $w(y)$ is the Hamming weight of y in d-ary representation, which is at least 1 for any $y > 0$. Hence, the minimum eigenvalue of $H_{in,t=0}|_{\mathcal{L}^\perp}$ is $1/(T+1)$. Since H_{in} consists of only positive semi-definite terms, we have

$$\lambda_1(H_{in}|_{\tilde{\mathcal{S}}_{prop}}) \geq \lambda_1(H_{in,t=0}|_{\tilde{\mathcal{S}}_{prop}}) \geq 1/(T+1). \tag{46}$$

Thus, to ensure that H_0 has spectral gap $\lambda_1(H_0|_{\mathcal{L}^\perp}) \geq 2\Delta$, we simply choose $J_{in} = O(\Delta(T+1))$, $J_{prop} = O(KT^2 J_{in}^2 m^2)$, and $J_{clock} = O(K J_{prop}^2 T^2) = O(\text{poly}(n, T, \Delta))$.

Now that we have shown H_0 has $\mathcal{L}$ as its groundspace with spectral gap 2Δ, we are ready to show that H_{circuit} (Δ, η, ϵ)-simulates H with only polynomial overhead in energy. To this end, we use the following result regarding perturbative reductions adapted from Lemma 4 of [8] (also Lemma 35 of [1]):

Lemma 4 (First-order reduction, adapted from [8])**.** *Suppose $\tilde{H} = H_0 + H_{out}$, defined on Hilbert space $\tilde{\mathcal{H}} = \mathcal{L} \oplus \mathcal{L}^\perp$ such that $H_0\mathcal{L} = 0$ and $\lambda_1(H_0|_{\mathcal{L}^\perp}) \geq 2\Delta$. Suppose H is a Hermitian operator and V is an isometry such that $\|VHV^\dagger - H_{out}|_{\mathcal{L}}\| \leq \epsilon/2$, then $\tilde{H}$ is a (Δ, η, ϵ)-simulation of H, as long as $\Delta \geq O(\epsilon^{-1}\|H_{out}\|^2 + \eta^{-1}\|H_{out}\|)$, per Definition 2. In other words, $\|\tilde{H}_{\leq\Delta} - \tilde{V}H\tilde{V}^\dagger\| \leq \epsilon$ for some isometry $\tilde{V}$ where $\|\tilde{V} - V\| \leq \eta$.* ♦

Observe we are given in the premise of this Lemma 1 that

$$\|VHV^\dagger - H_{out}|_{\mathcal{L}}\| \leq \epsilon/2. \tag{47}$$

Hence, $H_{\text{circuit}} = H_0 + H_{out}$ simulates H to precision (Δ, η, ϵ) where $\Delta \geq O(\epsilon^{-1}\|H_{out}\|^2 + \eta^{-1}\|H_{out}\|)$. The maximum interaction energy in H_{circuit} is $J_{clock} = O(\text{poly}(n, T, \Delta)) = O(\text{poly}(n, T, \epsilon^{-1}, \eta^{-1}, \|H_{out}\|))$. This concludes the proof of Lemma 1. □

We now prove the second Lemma, which shows that in order to ensure the circuit-Hamiltonian simulates the original Hamiltonian with good precision with trivial encoding, we only need to add $O(\text{poly}(n, \epsilon^{-1}))$ "idling" identity gates to the end of a polynomial-sized circuit before transforming the circuit back to a Hamiltonian.

Proof of Lemma 2. Note that we can write

$$|\eta_\mu\rangle = \sqrt{1-\chi^2}\, |\psi_\mu\rangle \otimes |\alpha\rangle + \chi\, |\beta_\mu\rangle \tag{48}$$

where

$$|\alpha\rangle = \frac{1}{\sqrt{L+1}} |0^m\rangle^{\text{anc}} \otimes \sum_{t=D}^{D+L} |1^t 0^{T-t}\rangle^{\text{clock}}, \tag{49}$$

$$|\beta_\mu\rangle = \frac{1}{\sqrt{D}} \sum_{t=0}^{D-1} \left(U_t \cdots U_2 U_1 |\psi_\mu\rangle |0^m\rangle^{\text{anc}} \right) |1^t 0^{T-t}\rangle^{\text{clock}}, \tag{50}$$

$$\text{and} \qquad \chi = \sqrt{D/(D+L+1)}. \tag{51}$$

Observe that $\langle \beta_\mu | (|\psi_\nu\rangle |\alpha\rangle) = 0$ since the clock register are at different times, and

$$\langle \beta_\mu | \beta_\nu \rangle = \frac{1}{D} \sum_{t=0}^{D-1} \langle \psi_\mu | \psi_\nu \rangle = \delta_{\mu\nu}. \tag{52}$$

Let $P_{\text{anc}} = |\alpha\rangle\langle\alpha|$. Then

$$\begin{aligned} H_{\text{eff}} - H \otimes P_{\text{anc}} &= \sum_\mu \Big[E_\mu |\eta_\mu\rangle\langle\eta_\mu| - E_\mu |\psi_\mu\rangle\langle\psi_\mu| \otimes |\alpha\rangle\langle\alpha| \Big] \\ &= \bigoplus_\mu \begin{pmatrix} -E_\mu \chi^2 & E_\mu \chi\sqrt{1-\chi^2} \\ E_\mu \chi\sqrt{1-\chi^2} & E_\mu \chi^2 \end{pmatrix} \end{aligned} \tag{53}$$

And so

$$\|H_{\text{eff}} - H \otimes P_{\text{anc}}\| \leq \chi \max_{\mu} E_{\mu} \leq \chi \|H\|. \tag{54}$$

To ensure $\|H_{\text{eff}} - H \otimes P_{\text{anc}}\| \leq \epsilon$, it's sufficient to choose L so that $\chi\|H\| = \epsilon$. Plugging in $\chi = \sqrt{D/(D+L+1)}$, we find that it is sufficient to choose $L = O(\frac{D\|H\|^2}{\epsilon^2})$.

□

B. Proof of Theorem 1 – Strongly Universal Hamiltonian on 2D Square Lattice

In this subsection, we show how to transform the spatially sparse Hamiltonian constructed previously into a Hamiltonian from a universal family of 2D spin-lattice model, with only polynomial overhead, proving our main Theorem 1. This follows from our Proposition 2 and the following result from Ref. [1]:

Lemma 5 (Essentially Ref. [1])**.** *Given any k-local n-qudit Hamiltonian H that is spatially sparse, we can construct H' from a family of $\mathcal{S}$-Hamiltonian on the 2D square lattice that efficiently (Δ, η, ϵ)-simulates H as long as $\mathcal{S}$ is non-2SLD. Here, $\Delta = O(\text{poly}(\|H\|, \eta^{-1}, \epsilon^{-1})$, and H' has $O(\text{poly}(n, \epsilon^{-1}))$ qubits and interaction energy $O(\text{poly}(\Delta))$.*

Then our main Theorem is a simple consequence of the above Lemma and our Proposition 2:

Proof of Theorem 1. As we showed in Proposition 2, any $O(1)$-local qudit Hamiltonian H with $\|H\|$ can be simulated by a spatially sparse 5-local Hamiltonian H_{circuit} with $O(\text{poly}(n)/\epsilon^2)$ terms and qubits, and interaction energy at most $O(\text{poly}(n, \eta^{-1}, \epsilon^{-1}))$. By Lemma 5, we can simulate H_{circuit} by a $\mathcal{S}$-Hamiltonian on the 2D square lattice with polynomial overhead, as long as $\mathcal{S}$ is non-2SLD. □

Although never formally stated, Lemma 5 is in fact one of the main results of Ref. [1]. Here we provide an outline of its proof for completeness.

Proof Outline of Lemma 5. To show this, we use a sequence of reductions originally described in Ref. [1, 5], which together performs the desired transformation. These reductions are enumerated in the following list of Lemmas:

Lemma 6 (Lemma 21 of [1])**.** *Given any k-local Hamiltonian on n qudits H, we can construct a $k\lceil\log_2 d\rceil$-local Hamiltonian H' on $n\lceil\log_2 d\rceil$ qubits that $(\Delta, 0, 0)$-simulates H, for $\Delta \geq \|H\|$. For $d = O(1)$, the construction preserves spatial sparsity, and uses terms of interaction energy $O(\Delta)$.*

The construction maps each qudit to $\lceil\log_2 d\rceil$ qubits, and uses local terms of strength Δ to penalize any redundant states among the qubits. Specifically, consider any isometry $W : \mathbb{C}^d \to (C^2)^{\otimes\lceil\log_2 d\rceil}$. The construction maps H to $H' = W^{\otimes n} H W^{\dagger\otimes n} + \Delta' \sum_{i=1}^{n} P_i$, for any $\Delta' > \Delta$, where $P = 1 - WW^\dagger$. It is easy to see that H' is spatially sparse if H is spatially sparse and $d = O(1)$.

Lemma 7 (Lemma 22 of [1])**.** *Given any k-local n-qubit Hamiltonian H, we can construct a real-valued $2k$-local $2n$-qubit Hamiltonian H' that $(\Delta, 0, 0)$-simulates H, for any $\Delta \geq 2\|H\|$. The construction preserves spatial sparsity, and uses terms of interaction energy $O(\Delta)$.*

The construction adds one additional qubit per original qubit, and map the individual Pauli operators in the following way:

$$\mathbb{1} \mapsto \mathbb{1} \otimes \mathbb{1}, \quad \sigma_{x,z} \mapsto \mathbb{1} \otimes \sigma_{x,z}, \quad \sigma_y \mapsto \sigma_y \otimes \sigma_y. \tag{55}$$

For each new pair of qubits $(i, n+i)$, an additional local term $\Delta'(Y_i Y_{n+i} + \mathbb{1})$ is added, where $\Delta' > \Delta$. Note the new Hamiltonian is real-valued, and spatially sparse if H is spatially sparse.

Lemma 8 (Lemma 39 of [1])**.** *Real-valued k-local qubit Hamiltonian H with M terms can be (Δ, η, ϵ)-simulated by real $(k+1)$-local Hamiltonian with $O(M+n)$ qubits and terms, whose Pauli-decomposition contains no Y terms. The construction preserves spatial sparsity, and uses interaction energy at most $\Delta = O(\text{poly}(\|H\|, \eta^{-1}, \epsilon^{-1}))$.*

The construction here takes any terms in H with (necessarily) even number of Y's, and recreates it with a perturbative gadget involving only X, Z terms and an additional mediator qubit a. Specifically, the gadget performs the following mapping:

$$Y^{\otimes 2m} \otimes A \quad \mapsto \quad \Delta h_0 + \sqrt{\Delta} h_2 \tag{56}$$

$$\text{where} \quad h_0 = (1 + Z_a)/2 = |0\rangle\langle 0|_a, \quad h_2 = X_a(X^{\otimes 2m} \otimes \mathbb{1} + (-1)^{m+1} Z^{\otimes 2m} \otimes A). \tag{57}$$

Every term is mapped in parallel with an independent mediator qubit gadget. Since there are M terms in H, we have at most $O(M + n)$ qubits in the end. The large interaction energy $\Delta = O(\mathrm{poly}(\|H\|, \eta^{-1}, \epsilon^{-1}))$ is required to ensure small errors from perturbation. It is easy to see that if H is spatially sparse, so is the new Hamiltonian.

Lemma 9 (Theorem 40 of [1]). *Suppose H is any k-local qubit Hamiltonian with M terms whose Pauli-decomposition contains no Y. Then H can be simulated by 2-local qubit Hamiltonians with $O(M + n)$ terms and qubits to precision (Δ, η, ϵ) whose Pauli-decomposition contains no Y terms. The construction preserves spatial sparsity, and uses interaction energy at most $\Theta(\Delta) = O(\mathrm{poly}(\|H\|, \eta^{-1}, \epsilon^{-1}))$ assuming $k = O(1)$.*

This construction makes use of the subdivision and 3-to-2 local gadgets, which are described in details in Ref. [5]. For each k-local term of the form $A \otimes B$, one can map it to $(\lceil k/2 \rceil + 1)$-local terms of the form $A \otimes X_w + X_w \otimes B$ using a subdivision gadget that introduces an extra ancilla qubit w. Thus, $O(\log k)$ applications of the subdivision gadget is sufficient to reduce the locality to 3-local. This is then reduced to 2-local terms using the 3-to-2 local gadget: this converts terms of the form $A \otimes B \otimes C$ to 2-local terms such as $(A - B)X_w$, $C|1\rangle\langle 1|_w$, AB, and $(A^2 + B^2)C$. In other words, the construction maps each k-local term to $O(k)$ 2-local terms mediated by $O(k)$ ancilla qubits. This mapping clearly preserves spatial sparsity. The required interaction energy blows up exponentially in k; however, since $k = O(1)$, the interaction energy required is at most $O(\mathrm{poly}(\|H\|, \eta^{-1}, \epsilon^{-1}))$.

Lemma 10 (Theorem 41 of [1]). *Suppose H is a 2-local n-qubit Hamiltonian whose Pauli-decomposition contains no Y terms. Then it can be simulated by a $4n$-qubit $\mathcal{S}$-Hamiltonian to precision (Δ, η, ϵ), where $\mathcal{S} = \{XX + YY + ZZ\}$ or $\{XX + YY\}$. The construction preserves spatial sparsity, and uses interaction energy at most $\Theta(\Delta) = O(\mathrm{poly}(\|H\|, \eta^{-1}, \epsilon^{-1}))$.*

Here, the construction uses a perturbative gadget that maps every logical qubit in H to a group of 4 physical qubits that interact only via terms from $\mathcal{S}$. The 1-local and 2-local interaction on any two logical qubits can be implemented using two-body terms from $\mathcal{S}$ coupling different pairs of physical qubits from the two groups. Hence, spatial sparsity is preserved by this construction. The required interaction energy scales as $\Theta(\Delta) = O(\mathrm{poly}(\|H\|, \eta^{-1}, \epsilon^{-1}))$.

Finally, we restate the following two results from Ref. [5]:

Lemma 11 (Lemma 46 of [1]). *Let $\mathcal{S}_0$ be either $\{XX + YY + ZZ\}$ or $\{XX + YY\}$. Any spatially sparse $\mathcal{S}_0$-Hamiltonian on n qubits, whose largest interaction energy is Λ_0, can be simulated by a $\mathcal{S}_0$-Hamiltonian on a 2D square lattice of $\mathrm{poly}(n)$ qubits using interaction energy at most $J_{ij} = O(\mathrm{poly}(n\Lambda_0(1/\epsilon + 1/\eta)))$.*

Lemma 12 (Theorem 42 of [1]). *Suppose $\mathcal{S}$ be a set of interactions on 2-qubits that is non-2SLD. Then given an $\{XX + YY + ZZ\}$- or $\{XX + YY\}$-Hamiltonian on the 2D square lattice, we can simulate it with an $\mathcal{S}$-Hamiltonian on the 2D square lattice.*

In what follows, we denote $\mathcal{S}_0$ as either $\{XX + YY + ZZ\}$ or $\{XX + YY\}$. For any $\mathcal{S}$ that is non-2SLD, we map H_{circuit} to an $\mathcal{S}$-Hamiltonian on the 2D square lattice in the following sequence:

1. By Lemma 6, we can simulate H_{circuit} with H_1 that is spatially sparse, $O(1)$-local on $O(\mathrm{poly}(n)/\epsilon^2)$ qubits and interaction energy at most $O(\|H_{\mathrm{circuit}}\|) = O(\mathrm{poly}(n, \eta^{-}1, \epsilon^{-}1))$.

2. By Lemma 7, we can simulate H_1 with H_2 that is spatially sparse, real-valued, and $O(1)$-local, with only polynomial overhead in qubit-number of interaction energy.

3. By Lemma 8, we can simulate H_2 with H_3 that is spatially sparse and $O(1)$-local, and contains no Y terms in Pauli-decomposition, with only polynomial overhead in qubit-number of interaction energy.

4. By Lemma 9, we can simulate H_3 with H_4 that is spatially sparse and 2-local, contains no Y terms in Pauli-decomposition, with polynomial overhead.

5. By Lemma 10, we can simulate H_4 with H_5 that is a spatially sparse $\mathcal{S}_0$-Hamiltonian, with polynomial overhead.

6. By Lemma 11, we can simulate H_5 by H_6 an $\mathcal{S}_0$-Hamiltonian on a 2D square lattice, with polynomial overhead.

7. By Lemma 12, we can simulate H_6 by the broader class of $\mathcal{S}$-Hamiltonian on the 2D square lattice, for any $\mathcal{S}$ that is non-2SLD, with polynomial overhead.

Since every step of the above sequence of reductions only incurs a polynomial overhead in the number of qubits and the strength of interactions, we have shown that any $O(1)$-local, polynomial-sized qudit Hamiltonians can be efficiently simulated by an $\mathcal{S}$-Hamiltonian with polynomial qubits and interaction energy, assuming $\mathcal{S}$ is non-2SLD. □

IV. SUPPLEMENTARY NOTE 4 – PROOF THAT 1D NEAREST-NEIGHBOR HAMILTONIANS ARE STRONGLY UNIVERSAL

Here we give our proof of Theorem 2, whose statement we reproduce below for convenience:

Theorem 2. *There is a strongly universal family of 1D Hamiltonians consisting of nearest-neighbor interactions acting on a line of 8-dimensional particles.*

The proof is based heavily on the framework in Ref. [9]. We will only try to provide a succinct and somewhat self-contained description of the most important elements of the construction here. For the full technical details of the construction, we encourage the reader to also examine Section 3 and 4 of Ref. [9].

A. Preliminaries

We first describe how the computation is encoded within a line of 8-dimensional particles.

Definition 5 (1D-encoded L-idling history state)**.** *Consider any quantum circuit U consisting of $R = O(\mathrm{poly}(n))$ rounds of 1-qubit or nearest-neighbor 2-qubit gates on a line of n qubits. This can be further converted to an* encoded circuit *$\tilde{U}$ with nearest-neighbor gates acting on a line $2nR + L$ qudits ($d = 8$), implicitly arranged in R blocks of $2n$ qudits, followed by L qudits for idling. The Hilbert space of each qudit is $\mathcal{H}_8 = \bigcirc \oplus \ominus \oplus \circledcirc \oplus \otimes \oplus \square \oplus \boxed{\blacktriangleright}$, where $\square$ and $\boxed{\blacktriangleright}$ are 2-dimensional subspaces designed to hold a qubit state, and the rest are 1-dimensional subspaces. For a given input state of the form $|\psi_\mu\rangle |0^m\rangle \in \mathbb{C}^{2^n}$ on the original n qubits, this is encoded as*

$$|\gamma_0^\mu\rangle = \Vert \overbrace{\underbrace{\boxed{\blacktriangleright}\circledcirc|\square\circledcirc|\cdots|\square\circledcirc|\square\bigcirc}_{\text{the first block of length } 2n}\Vert\bigcirc\bigcirc|\bigcirc\bigcirc|\cdots\cdots}^{R\text{ blocks}}\bowtie\underbrace{\bigcirc\bigcirc\bigcirc\cdots\bigcirc}_{L\text{ idling qudits}} \tag{58}$$

where the odd qudits in the first block of length $2n$ encodes the input state $|\psi_\mu\rangle |0^m\rangle$. Here, the symbols $\Vert$, $|$ and $\bowtie$ are simply boundary markers in space that help us identify the role of each particle and do not indicate anything about the internal state of particles. In particular, the symbol $\bowtie$ marks a special boundary that separates the computational part of the line and the idling part.

The encoded circuit $\tilde{U}$ acts on $|\gamma_0^\mu\rangle$ with R rounds of computation, each corresponding to applying a round of gates from U' to the currently active block of qudits, and then moving the block $2n$ positions to the right. This entails a total of $K = (R-1)(3n^2+2n-1)+2n$ steps of nearest-neighbor gates that map configuration to configuration, according to the transition rule outlined in Table 1 of Ref. [9]. This is then followed by L steps of "idling" where the L rightmost qudits perform a trivial counting operation. To facilitate the idling, we add the following transition rules.

- *(α) $\boxed{\blacktriangleright}\bowtie\bigcirc \longleftrightarrow \square\bowtie\otimes$ unmarks the active qubit $\boxed{\blacktriangleright}$ once it hits the special boundary $\bowtie$. The $\bigcirc$ changes to $\otimes$ so as to signal that the idling is supposed to the start.*
- *(β) $\otimes\bigcirc \longleftrightarrow \otimes\otimes$ for any location to the right of the special boundary $\bowtie$.*

This results in a history of $K + L + 1$ configurations on the $2nR$ qudits $\{|\gamma_t^\mu\rangle\}_{t=0}^{K+L}$ which is pairwise orthogonal: $\langle\gamma_t^\mu|\gamma_{t'}^\mu\rangle = \delta_{tt'}$. Finally, we define the 1D-encoded L-idling history state *with respect to U and $|\psi_\mu\rangle |0^m\rangle$ as the following superposition state:*

$$|\eta_\mu\rangle = \frac{1}{\sqrt{K+L+1}} \sum_{t=0}^{K+L} |\gamma_t^\mu\rangle\,. \tag{59}$$

To help visualize the computational history according to the transition rules in Ref. [9], we remark that the configuration after all R rounds of computation comes to halt is

$$|\gamma_K^\mu\rangle = \|\otimes^{2n(R-1)}\|\otimes\Box|\circledcirc\Box|\cdots|\circledcirc\Box|\circledcirc\boxed{\blacktriangleright}\bowtie\bigcirc^{\otimes L}. \tag{60}$$

All ensuing configurations are of the form (for $1 \le \ell \le L$):

$$|\gamma_{K+\ell}^\mu\rangle = \|\otimes^{2n(R-1)}\|\otimes\Box|\circledcirc\Box|\cdots|\circledcirc\Box|\circledcirc\Box\bowtie\otimes^{\otimes\ell}\bigcirc^{\otimes(L-\ell)}. \tag{61}$$

Lemma 13 (1D circuit-Hamiltonian [9])**.** *Given a circuit U consisting of* poly(n) *1- or 2-qubit gates on n qubits. We can construct a Hamiltonian on $O(\mathrm{poly}(n)) + L$ qudits, $d = 8$, with only nearest-neighbor interaction of the form*

$$H_{\rm hist} = J_{\rm in}H_{\rm in} + J_{\rm prop}H_{\rm prop} + J_{\rm pen}H_{\rm pen} \tag{62}$$

such that the 1D-encoded L-idling history states with respect to U and $|\psi_\mu\rangle|0^m\rangle$ are of zero-energy, and all other states have energy ≥ 1. The interaction energy of nearest-neighbor terms in $H_{\rm hist}$ are $J_{\rm in}, J_{\rm prop}, J_{\rm pen} = O(\mathrm{poly}(n))$.

Proof. The construction is essentially the same as described in Section 4 of Ref. [9], except for a few small changes:

a. Changes to legal configurations and penalty Hamiltonian — To the right of the special boundary $\bowtie$, only $\otimes\otimes$, $\otimes\bigcirc$ and $\bigcirc\bigcirc$ are allowed configurations in these locations. This can be addressed by tweaking the penalty Hamiltonian $H_{\rm pen}$ to penalize all configurations using the term $|XY\rangle\langle XY|_{i,i+1}$ where $XY \in \mathcal{H}_8^{\otimes 2}\backslash\{\otimes\otimes, \otimes\bigcirc, \bigcirc\bigcirc\}$ for these locations.

b. Changes to propagation Hamiltonian — We need to incorporate the two new rules (α) and (β) added above. The Rule (α) is similar to Rule 4a $\boxed{\blacktriangleright}\|\bigcirc \longleftrightarrow \Box\|\ominus$ from Table 1 of Ref. [9]. Since there's only a unique location where Rule (α) applies, at the special boundary, we can simply use the following propagation Hamiltonian for that pair of sites

$$H^{(\alpha)}_{{\rm prop},i} = |\boxed{\blacktriangleright}\bowtie\bigcirc\rangle\langle\boxed{\blacktriangleright}\bowtie\bigcirc|_{i,i+1} + |\Box\bowtie\otimes\rangle\langle\Box\bowtie\otimes|_{i,i+1} - |\Box\bowtie\otimes\rangle\langle\boxed{\blacktriangleright}\bowtie\bigcirc|_{i,i+1} - |\boxed{\blacktriangleright}\bowtie\bigcirc\rangle\langle\Box\bowtie\otimes|_{i,i+1} \tag{63}$$

where $i = 2nR$ for this special pair of sites.

Now for Rule (β). Note this is the only propagation rule that is applicable in the region to the right of the special boundary $\bowtie$. Thus, we only need the following propagation Hamiltonian for $i > 2nR$.

$$H^{(\beta)}_{{\rm prop},i} = |\otimes\bigcirc\rangle\langle\otimes\bigcirc|_{i,i+1} + |\otimes\otimes\rangle\langle\otimes\otimes|_{i,i+1} - |\otimes\otimes\rangle\langle\otimes\bigcirc|_{i,i+1} - |\otimes\bigcirc\rangle\langle\otimes\otimes|_{i,i+1} \tag{64}$$

We note there can be mis-timed transitions from $H^{(\alpha)}_{{\rm prop},i}$, e.g.

$$\circledcirc\Box\bowtie\otimes\otimes \quad\longrightarrow\quad -\circledcirc\boxed{\blacktriangleright}\bowtie\bigcirc\otimes \tag{65}$$

and $H^{(\alpha)}_{{\rm prop},i}$, e.g.,

$$\otimes\otimes\otimes \quad\longrightarrow\quad -\otimes\bigcirc\otimes \tag{66}$$

However, they will all result in energy penalty from $H_{\rm pen}$, because they have illegal configuration $\bigcirc\otimes$ that is locally detectable.

c. Proof that the Hamiltonian has the 1D-encoded history states as the only ground states — This is essentially given in Section 5 and 6 of [9]. □

B. Proof of Theorem 2

Proof of Theorem 2. As in the Proof of Theorem 1, we can always convert any input qudit Hamiltonian to a qubit Hamiltonian by encoding each qudit in a group of $\lceil\log_2 d\rceil$-qudits with polynomial overhead (assuming $d = O(1)$). Hence, we will take the input H as an $O(1)$-local n-qubit Hamiltonian. We write $H = \sum_\mu E_\mu|\psi_\mu\rangle\langle\psi_\mu|$ in its eigenbasis, with $0 \le E_\mu \le E_{\rm max}$. By Proposition 1, we can construct a circuit $U^{\rm NN}_{\rm PE}$ consisting on $O(\mathrm{poly}(n, \zeta^{-1}))$ 1- or 2-qubit nearest-neighbor gates on $n+m$ qubits, $m = O(\mathrm{poly}(n))$, such that its action on any normalized state $\sum_\mu c_\mu|\psi_\mu\rangle$ can be described as

$$\left\| U^{\rm NN}_{\rm PE}\sum_\mu c_\mu|\psi_\mu\rangle|0^m\rangle - \sum_\mu c_\mu|\psi_\mu\rangle|\tilde{E}_\mu\rangle|{\rm rest}_\mu\rangle\right\| \le \zeta \tag{67}$$

where $|\tilde{E}_\mu\rangle = |\varphi_{\mu,1}\varphi_{\mu,2}\varphi_{\mu,3}\ldots\varphi_{\mu,s}\rangle$ is the s-bit string representation of $\varphi_\mu = E_\mu/E_{\max} = 0.\varphi_{\mu,1}\varphi_{\mu,2}\varphi_{\mu,3}\cdots$. From now on we will denote $\tilde{n} = n+m$ as the number of qubits in the phase estimation circuit.

We then consider the identity circuit $U = (U_{\mathrm{PE}}^{\mathrm{NN}})^\dagger \mathbb{1}^{n+m} U_{\mathrm{PE}}^{\mathrm{NN}} = \mathbb{1}$ that corresponds to running $U_{\mathrm{PE}}^{\mathrm{NN}}$, applying identity to every qubit, and then uncomputing. Let R_{PE} be the number of rounds of nearest-neighbor gates in U_{PE}, and $R = 2R_{\mathrm{PE}}+1$ be the total number of rounds. Let $K = (R-1)(3\tilde{n}^2+2\tilde{n}-1)+2\tilde{n}$ be the total number of steps when applying U. By Lemma 13, we can construct a 1D nearest-neighbor Hamiltonian H_{hist} on $\mathrm{poly}(n)+L$ particles with $\mathrm{poly}(n)$ interaction energy such that all the 1D-encoded L-idling history states with respect to U and $|\psi_\mu\rangle|0^m\rangle$ are the only zero-energy states, and all other states have energy at least Δ.

We claim that the following 1D Hamiltonian (Δ, η, ϵ)-simulates H

$$\tilde{H}_{\mathrm{1D}} = 2\Delta H_{\mathrm{hist}} + H_{\mathrm{out}}. \tag{68}$$

To describe H_{out}, we start by considering the configuration of all $2\tilde{n}R+L$ particles after the R_{PE}-th round of gates corresponding to the end of applying $U_{\mathrm{PE}}^{\mathrm{NN}}$. This looks like

$$\cdots \otimes \otimes \| \boxed{\blacktriangleright} \circledcirc | \square \circledcirc | \cdots \square \circledcirc | \square \bigcirc \| \bigcirc \bigcirc \cdots \bigcirc \bowtie \bigcirc \cdots \tag{69}$$

Note the next round of gates corresponds to $\mathbb{1}^{n+m}$, i.e., applying the identity gate to every qubit. Without loss of generality, we assume the first s active particles corresponds (ζ-approximately) to the ancilla with $|\tilde{E}_\mu\rangle$ on them. Notice that every active qudit will at one point transition into their appropriate state in the $\boxed{\blacktriangleright}$ subspace, with identity gate applied. Then, the appropriate H_{out} is

$$H_{out} = (K+L+1)E_{\max}\sum_{b=1}^{s} 2^{-b} |\boxed{\blacktriangleright}^{(1)}\rangle\langle\boxed{\blacktriangleright}^{(1)}|_{2\tilde{n}R_{\mathrm{PE}}+2b-1} \tag{70}$$

where $|\boxed{\blacktriangleright}^{(1)}\rangle$ corresponds to the $|1\rangle$ qubit state in the $\boxed{\blacktriangleright}$ subspace.

To prove that $\tilde{H}_{\mathrm{1D}}$ simulates H to the desired precision, we first show that H_{out} restricted to the set of 1D-encoded history states $\mathcal{L} = \mathrm{span}\{|\eta_\mu\rangle\}$ can be approximated by the following effective Hamiltonian:

$$H_{\mathrm{eff}} = \sum_\mu E_\mu |\eta_\mu\rangle\langle\eta_\mu| \tag{71}$$

Consider arbitrary states $|\eta\rangle \in \mathcal{L}$. We write $|\eta\rangle = \sum_\mu a_\mu |\eta_\mu\rangle$, and observe

$$\begin{aligned}\langle\eta|H_{out}|\eta\rangle &= (K+L+1)E_{\max}\sum_{b=1}^{s} 2^{-b}\sum_{\nu,\mu} a_\nu^* a_\mu \langle\eta_\nu| \left(|\boxed{\blacktriangleright}^{(1)}\rangle\langle\boxed{\blacktriangleright}^{(1)}|_{2\tilde{n}R_{\mathrm{PE}}+2b-1}\right)|\eta_\mu\rangle \\ &= E_{\max}\sum_{b=1}^{s} 2^{-b}\sum_{\nu,\mu}\sum_{t,t'=0}^{K} a_\nu^* a_\mu \langle\gamma_t^\nu| \left(|\boxed{\blacktriangleright}^{(1)}\rangle\langle\boxed{\blacktriangleright}^{(1)}|_{2\tilde{n}R_{\mathrm{PE}}+2b-1}\right)|\gamma_{t'}^\mu\rangle \end{aligned} \tag{72}$$

Note the $2\tilde{n}R_{\mathrm{PE}}+2b-1$-th particle is only in the $\boxed{\blacktriangleright}$ subspace at one point in time among the $K+1$ steps, regardless of possible input state. Table 2 of [9] provides a clear illustration for the reason. Let's call that time t_b, and write

$$\langle\eta|H_{out}|\eta\rangle = E_{\max}\sum_{b=1}^{s} 2^{-b}\sum_{\nu,\mu} a_\nu^* a_\mu \langle\gamma_{t_b}^\nu| \left(|\boxed{\blacktriangleright}^{(1)}\rangle\langle\boxed{\blacktriangleright}^{(1)}|_{2\tilde{n}R_{\mathrm{PE}}+2b-1}\right)|\gamma_{t_b}^\mu\rangle \tag{73}$$

Note the configuration $\sum_\mu a_\mu |\gamma_{t_b}^\mu\rangle$ is the encoded version of $U_{\mathrm{PE}}^{\mathrm{NN}}\sum_\mu a_\mu |\psi_\mu\rangle|0^m\rangle$, which is ζ-close to the encoded version of $\sum_\mu a_\mu |\psi_\mu\rangle|\tilde{E}_\mu\rangle|\mathrm{rest}_\mu\rangle$. Hence

$$\begin{aligned}\langle\eta|H_{out}|\eta\rangle &= E_{\max}\sum_{b=1}^{s} 2^{-b}\sum_{\nu,\mu} a_\nu^* a_\mu \varphi_{\mu,b}\langle\psi_\nu|\psi_\mu\rangle\langle\mathrm{rest}_\nu|\mathrm{rest}_\mu\rangle + \Theta(\zeta E_{\max}) \\ &= \sum_\mu |a_\mu|^2 \sum_{b=1}^{s} 2^{-b}\varphi_{\mu,b}E_{\max} + \Theta(\zeta E_{\max}) \\ &= \sum_\mu |a_\mu|^2 \tilde{E}_\mu + \Theta(\zeta E_{\max}). \end{aligned} \tag{74}$$

where we have denoted $\tilde{E}_\mu = E_{\max}\tilde{\varphi}_\mu = E_{\max}(0.\varphi_{\mu,1}\varphi_{\mu,2}\cdots\varphi_{\mu,s})$ as the s-bit representation of energy eigenvalue E_μ of H. Then

$$|\langle\eta|H_{out} - H_{\text{eff}}|\eta\rangle| \le \sum_\mu |a_\mu|^2 |\tilde{E}_\mu - E_\mu| + \Theta(s\zeta E_{\max}) \le [2^{-s} + \Theta(\zeta)]E_{\max} \tag{75}$$

By choosing $s = \Theta(\log E_{\max}/\epsilon) = \Theta(\log n + \log \epsilon^{-1})$ and $\zeta = \Theta(\epsilon/E_{\max}) = \Theta(1/\operatorname{poly}(n, \epsilon^{-1}))$, just like we did in Eq. (31), we can ensure

$$|\langle\eta|H_{out} - H_{\text{eff}}|\eta\rangle| \le \epsilon/4 \quad \forall\,|\eta\rangle \in \mathcal{L} \quad\implies\quad \|H_{\text{eff}} - H_{out}|_{\mathcal{L}}\| \le \epsilon/4 \tag{76}$$

Furthermore, looking at the 1D-encoded L-idling history state more carefully, it looks like

$$|\eta_\mu\rangle = \frac{1}{\sqrt{K+L+1}} \sum_{t=0}^{K+L} |\gamma_t^\mu\rangle = \sqrt{1-\chi^2}\,|\alpha_\mu\rangle + \chi\,|\beta_\mu\rangle \tag{77}$$

where

$$\chi = \sqrt{\frac{K+1}{K+L+1}} \tag{78}$$

and

$$|\beta_\mu\rangle = \frac{1}{\sqrt{K+1}} \sum_{t=0}^{K} |\gamma_t^\mu\rangle \tag{79}$$

$$\begin{aligned} |\alpha_\mu\rangle &= \frac{1}{\sqrt{L}} \sum_{t=1}^{L} |\gamma_{K+t}^\mu\rangle = \|\otimes^{2\tilde{n}(R-1)}\|\otimes\square|\circledcirc\square|\cdots|\circledcirc\square|\circledcirc\square\bowtie \frac{1}{\sqrt{L}} \sum_{\ell=1}^{L} \otimes^{\otimes\ell} \bigcirc^{\otimes(L-\ell)} \\ &= (V\,|\psi_\mu\rangle) \otimes |\alpha\rangle \end{aligned} \tag{80}$$

where $V\,|\psi_\mu\rangle$ is simply the input state $|\psi_\mu\rangle$ stored in the qubit subspace of a subset of the $\tilde{n}$ qudits marked with $\square$ in the R-th block, $|\alpha\rangle$ is the state of the remaining ancilla. Therefore,

$$|\eta_\mu\rangle = \sqrt{1-\chi^2}(V\,|\psi_\mu\rangle) \otimes |\alpha\rangle + \chi\,|\beta_\mu\rangle\,. \tag{81}$$

By choosing $L = O(K/\epsilon^2)$, we can ensure that $\||\eta_\mu\rangle\langle\eta_\mu| - V|\psi_\mu\rangle\langle\psi_\mu|V^\dagger \otimes |\alpha\rangle\langle\alpha|\| \le O(\epsilon)$. With the same argument in Lemma 2, we can show that

$$\|H_{\text{eff}} - VHV^\dagger \otimes |\alpha\rangle\langle\alpha|\| \le \epsilon/4 \tag{82}$$

Therefore, together with Eq. (76) we have

$$\left\|VHV^\dagger \otimes |\alpha\rangle\langle\alpha| - H_{out}|_{\mathcal{L}}\right\| \le \epsilon/2 \tag{83}$$

To finish proving our Theorem 2, we again use Lemma 4 which we restate below for the reader's convenience:

Lemma 4 (First-order reduction, adapted from [8])**.** *Suppose $\tilde{H} = H_0 + H_1$, defined on Hilbert space $\tilde{\mathcal{H}} = \mathcal{L} \oplus \mathcal{L}^\perp$ such that $H_0\mathcal{L} = 0$ and $\lambda_1(H_0|_{\mathcal{L}^\perp}) \ge 2\Delta$. Suppose H is a Hermitian operator and V is an isometry such that $\|VHV^\dagger - H_1|_{\mathcal{L}}\| \le \epsilon/2$, then $\tilde{H}$ (Δ, η, ϵ)-simulates H, as long as $\Delta \ge O(\epsilon^{-1}\|H_1\|^2 + \eta^{-1}\|H_1\|)$, per Definition 2. In other words, $\|\tilde{H}_{\le\Delta} - \tilde{V}H\tilde{V}^\dagger\| \le \epsilon$ for some isometry $\tilde{V}$ where $\|\tilde{V} - V\| \le \eta$.*

We apply the above Lemma with $H_0 = 2\Delta H_{\text{hist}}$ and $H_1 = H_{out}$. Thus, $\tilde{H}_{\text{1D}} = H_0 + H_1$ simulates H to precision (Δ, η, ϵ) by choosing $\Delta \ge O((\epsilon^{-1} + \eta^{-1})\operatorname{poly}(n, \|H\|))$. Note that $\tilde{H}_{\text{1D}}$ contains $O(\operatorname{poly}(n, \epsilon^{-1}))$ nearest-neighbor terms, with interaction energy $O(\operatorname{poly}(n, \eta^{-1}, \epsilon^{-1}, \Delta))$, which proves our Theorem. $\square$

---

[1] Cubitt, T. S., Montanaro, A. & Piddock, S. Universal quantum Hamiltonians. *Proceedings of the National Academy of Sciences* **115**, 9497–9502 (2018).

[2] Nielsen, M. A. & Chuang, I. L. *Quantum Computation and Quantum Information* (Cambridge University Press, New York, NY, USA, 2011), 10th edn.
[3] Dawson, C. M. & Nielsen, M. A. The Solovay-Kitaev Algorithm. *Quantum Info. Comput.* **6**, 8195 (2006).
[4] Kitaev, A. Y., Shen, A. & Vyalyi, M. N. *Classical and Quantum Computation* (American Mathematical Society, 2002).
[5] Oliveira, R. & Terhal, B. M. The complexity of quantum spin systems on a two-dimensional square lattice. *Quantum Inf. Comput.* **8**, 900–924 (2008).
[6] Aharonov, D. *et al.* Adiabatic quantum computation is equivalent to standard quantum computation. *SIAM J. Comput.* **37**, 166–194 (2007).
[7] Kempe, J., Kitaev, A. & Regev, O. The Complexity of the Local Hamiltonian Problem. *SIAM J. Comput.* **35**, 1070–1097 (2006).
[8] Bravyi, S. & Hastings, M. On complexity of the quantum ising model. *Commun. Math. Phys.* **349**, 145 (2016).
[9] Hallgren, S., Nagaj, D. & Narayanaswami, S. The Local Hamiltonian Problem on a Line with Eight States is QMA-complete. *Quantum Info. Comput.* **13**, 721–750 (2013).
