# Supplementary material for: Universal Hamiltonian simulators in one and two dimensions
Source: Nat Commun. 2026 Apr 21;17:6888. doi: 10.1038/s41467-026-71686-4 (PMC13388968; doi:10.1038/s41467-026-71686-4)
Supplement: Supplementary file 2 — Transparent Peer Review file [file 41467_2026_71686_MOESM2_ESM.pdf]

nature portfolio

Peer Review File

Universal Hamiltonian Simulators

Corresponding Author: Professor Leo Zhou

This file contains all reviewer reports in order by version, followed by all author rebuttals in order by version.

Version 0:

Reviewer comments:

Reviewer #1

(Remarks to the Author)
The main result of this work is what the authors call a “strongly universal simulation” of O(1)-local Hamiltonians using local Hamiltonians on 2D and 1D grids. In the case of reduction to 2D, the resulting Hamiltonian can be composed of Heisenberg or XY interactions alone. In contrast to “weakly universal simulation” constructions given in previous works, the “strong” version has only a polynomial overhead in the number of particles or interaction energy. This has the conceptual significance of removing all exponential overheads from the simulation, which also brings it closer to experimental reality.

To construct their “strongly universal Hamiltonians”, the authors draw from an arsenal of techniques in Hamiltonian complexity theory. The key first step is to consider the phase estimation circuit for the Hamiltonian which is the target for simulation. For O(1)-local Hamiltonians, such a circuit can then be brought down to a 2D or even 1D locally sparse form, using known techniques in complexity theory. The resulting circuit can in turn be mapped via a circuit-to-Hamiltonian construction to a locally sparse history Hamiltonian, with extra penalties for the phase-estimated energy. The authors prove that this locally-sparse Hamiltonian simulates the original one, penalty terms reproducing the spectrum of the original Hamiltonian. Conversion to a 2D square grid with a single type of interaction (e.g., Heisenberg), is done in the same way as in the earlier “weak Hamiltonian simulation” result by Cubitt, Montanaro and Piddock. Unlike in the latter work, the constructed Hamiltonian suffers no exponential overhead in energy, thanks to the history Hamiltonian constructed by the authors already being locally sparse in 2D. This also shows that the family of weakly universal Hamiltonians of Cubitt et al is in fact strongly universal.

In addition to the key result, the authors analyzed a variety of important auxiliary questions. For example, they pin down the precise relation between the subtly different concepts of “dynamics” and “analog” simulation. Also given is the proof of 2D strongly universal Hamiltonians for interactions of “non-2SLD” type, which generalizes the Heisenberg and XY options. Furthermore, the authors discuss possible extension of their results to fully translationary invariant Hamiltonians, and the implications of their work for simulations of Hamiltonians beyond O(1)-local (e.g., via quantum Church-Turing thesis).

The problem of “strong simulability” by 1D and 2D Hamiltonians, addressed by the authors, is significant conceptually as well as for experimental realizations of analog quantum simulation. The problem is nontrivial, and was even regarded as potentially unsolvable in the paper of Cubitt et al. To solve it, the authors had to draw on a variety of complexity-theoretic ideas in an original and technically sophisticated way. The authors’ analysis of dynamics vs. analog simulation and other auxiliary questions are also very welcome additions to the field. Finally, I find the very fact of successfully applying complexity-theoretic ideas to realistic analog quantum simulation a remarkable achievement in theoretical physics and computer science.

One minor thing I felt is missing is a concrete discussion about the possible next steps in making such an analog simulation realistic. Of course, a satisfactory analysis of the precise costs involved in implementing the authors’ protocol would constitute a major work in itself; improving these costs further is a clearly promising line of future research. However, a brief discussion of these aspects would be a welcome addition to the field, given that many practically minded people are alien to complexity theoretic techniques. In particular, the reader may be interested in the authors’ expert opinion on

(1) Which transformations in their construction should be revisited first, if one wants to improve the scaling with n and error, as well as the prefactor constants, in the particle count and prefactor energies? How much can it be improved? (I find it already useful that the authors comment on impossibility of attaining constant overhead in energies; having more of such insights in writing would be useful)

(2) Which hardware may be best suited for simulation? (given the peculiarity of high energy overheads, high qubit counts, high control requirements, etc)

(3) Which imperfections in hardware should one be most worried about? (e.g. stray or uncontrolled interactions - which strength, which type)

(4) Which scaling of the prefactor energies may be satisfactory from realistic standpoint (if polynomial, then which degree, etc), and if there are any strong no-go statements about achieving practical analog simulation via such a universal reduction

(5) Which trade-offs exist between different requirements on the simulating Hamiltonian, which are useful to keep in mind for applications? (e.g., if one is happy doing non-semi-TI physics, then one saves qubits and energy on mapping to Heisenberg; maybe some concrete families are cheaper to simulate than a general O(1)-local H; etc)

Comments which would shed light on any of the above questions would benefit the paper and may greatly assist future research. I leave it up to the authors which exact points and to which extent they choose to elaborate on.

By the way, please see a minor typo – "contains no any Y terms", supplement p. 10.

To conclude, I enthusiastically recommend this paper for acceptance in Nature communications. Furthermore, I found only a very limited need for revisions (as described above). The manuscript is exceptionally well-written and well-structured, explaining the result on multiple levels of detail. Having carefully examined the work including the Supplemental Material p.II-IV, I found no logical flaw in the authors' argument. Most questions I had were answered in the text in a very clear way.

I sincerely congratulate the authors on attaining these great results, and express my gratitude as a reader for writing an excellent manuscript.

Reviewer #2

(Remarks to the Author)
Summary and main results:
This paper is about analog simulations of quantum systems, which is where a quantum system is constructed so that its low energy space reproduces a different target system (up to some encoding).
This is based on the definition from ref [16] which also showed that certain families of Hamiltonians are universal in the sense that they can simulate all other Hamiltonians. Previous constructions included qubit simulator Hamiltonians restricted to 2D grids [16] and were extended in [18,19] to translationally invariant Hamiltonians in 2D and 1D. But the previous constructions with restricted spatial locality had interaction strengths that scaled exponentially with the size of the target system, and so were deemed inefficient.

The main contribution of this paper is efficient (ie poly sized interaction strengths) universal simulator constructions for 2D qubit and 1D qudit (8 dimensional subsystems) Hamiltonians, which are not translationally invariant. It is also shown that efficiently universal Hamiltonians can efficiently simulate any Hamiltonian whose time dynamics can be efficiently simulated. Finally there is a comparison to a different definition of dynamics simulation, and it is shown that the definition of analog simulation from [16] is stronger.

Correctness:
The results all seem to be correct as far as I have checked them and are presented rigorously in the Supplementary Information, although I have not checked all of this. The detailed proof sketches in the Methods appendix are clear enough descriptions of the proofs (and how they work using previous methods combined in a logical way) that I have good confidence all of the results are correct.

Proof methods:
The proofs of the constructions in 2D and 1D are both based on an idea from [21] by the same authors. It involves a very clever combination of standard quantum computing techniques: convert the target Hamiltonian into a circuit for dynamics simulation, then do phase estimation on this circuit, and convert back to a Hamiltonian using Kitaev's history state construction. Adding penalty terms to get the right energy for each state, and an idling trick to get a local encoding, completes the proof. Simple tricks for sparsifying the intermediate circuit results in a sparse simulator Hamiltonian.
This powerful idea provides an easy way to generate new analog simulator constructions (by replacing the last step with a different circuit-to-Hamiltonian construction), and this crudely describes the approach taken in [19] and here.

There are a couple of extra tricks used here: for the 2D result, the circuit is transformed into a 1D nearest neighbour circuit, then using SWAP gates to a spatially sparse 2D circuit where the second dimension represents time. This results in a circuit which is spatially sparse so that the standard techniques of ref [27] can be used to convert to a 2D simulator Hamiltonian.
For the 1D result, the circuit-to-Hamiltonian construction of [29] is used but here the idling trick would result in a highly non-local encoding. This is circumvented by a simple modification to the transition rules so that the idling can be done "in place".

To summarise my comments on proof methods: the main ideas largely come from other papers, but they are combined in a non-trivial way to get the genuinely new results, and there are other technical challenges which are neatly overcome with

some simple tricks.

Significance of results:
Analog simulators could be a practical use of small quantum computers or systems without error correction, and it is promising that previous work showed that simple qubit interactions can be universal. However for these simulators to be useful they need to be as close to practically realisable as possible. Previous constructions had the major drawback of either having interaction strengths growing exponentially with system size, or having long range interactions. The improvement here to efficient universal simulators in 2D therefore represents a major step forward in our understanding of analog simulators (with respect to the strong definition of [16]) and to their potential practical application.
Overall I liked the results, and I found the paper clear and well-written, so I would recommend acceptance.

Other comments:
I realise the first draft of this paper appeared on the arXiv in Feb 2021, and the literature review is generally very good, but I'd be interested if the authors had some brief comments on this paper https://arxiv.org/abs/2101.12319. It came on the arXiv at about the same time (just before), and has some general conditions for analog simulations to be efficient and universal – can these conditions be easily checked for the 2D and 1D cases in this paper?

I am not convinced that you need to introduce the new definitions of "strong simulation" and "weak simulation", when the same idea (as I understand it) was previously covered by "efficient" and "inefficient". I realise that efficiency is very important for implementations of simulators, and that calling it strong/weak emphasises this point, but I feel introducing extra terminology may be unnecessarily confusing, so I'd ask the authors to consider sticking to efficient/inefficient?

Typo beneath equation (9) of supplementary information: implmentation.

Reviewer #3

(Remarks to the Author)
Overview

This paper considers the notion of analog Hamiltonian simulation — that is, simulating, or reproducing the physics of, a target Hamiltonian by implementing another 'simulator' Hamiltonian. In a previous work, Cubit, Montanaro and Piddock [CMP19] formalized the theory of analog Hamiltonian simulation, and demonstrated the existence of 'universal' families of Hamiltonians — those that can simulate any target Hamiltonian. Remarkably, it turned out that many simple families of Hamiltonian, such as the Heisenberg or XY model on a square lattice, are instances of such universal Hamiltonians.

Such results are both theoretically interesting, having been used to prove various results in quantum complexity theory, as well as practically relevant: they open the door to simulating a wide range of quantum mechanical systems via the implementation of a chosen universal Hamiltonian, a use-case that covers one of the most important potential applications of quantum computers, and possibly without requiring a fault-tolerant gate-based device. Unfortunately, the aforementioned universality results came with a drawback: the simulations are only efficient for 1D and 2D target Hamiltonians. For more general target Hamiltonians, the simulators require resources growing exponentially with system size.

In this paper, the authors overcome this drawback, and demonstrate that the universal families of Hamiltonians considered in that work (and follow-up works), as well as a new 1D simulator Hamiltonian, can in fact simulate all target local Hamiltonians with only a polynomial increase in resources. They call this 'strong universal simulation' to distinguish it from the 'weak universal simulation' of previous results. This could give rise to new theoretical results (indeed a previous version of this paper has already yielded new results in some complexity theory works), as well as opening the door to efficient analog simulation of important Hamiltonians that are not 1D or 2D (such as the electronic structure Hamiltonians appearing in chemistry).

Results

The authors use a different approach to previous works in this area to show that all Hamiltonians that are efficiently simulable via quantum circuits (in the sense of implementing time evolution) can be efficiently strongly simulated by a range of 2D and 1D families of Hamiltonians. The 2D families were already known (to be *weak* universal simulators), whereas the 1D family is new. Specifically, the main results shown in this paper are:

The existence of strongly universal families of 2D Hamiltonians. These are so-called semi-translationally invariant (meaning that the interactive *terms* in the Hamiltonian are identical across sites, but the interaction *strengths* are not). This is in contrast to some known (fully-)translationally invariant weakly universal families, though one probably cannot expect to recover full translation-invariance for strong universality.
The existence of a strongly universal family of 1D Hamiltonians (on 8-dimensional audits, not (semi-)translationally invariant).

The authors also consider the notion of dynamics simulation: using the time-dynamics of a simulator Hamiltonian to simulate

the time-dynamics of a target Hamiltonian. The authors show that analog simulation (in the sense defined by [CMP19]) is a strictly stronger condition than dynamics simulation. In particular, it is shown that every analog simulator is a 'dynamics' simulator (with some appropriate definition thereof), but that the converse does not necessarily always (fully) hold. Strong universality for dynamics simulation has been shown in previous works, and so this fact separates the contribution of the current work with those.

Techniques

For the first result, the main steps of the proof are i) to construct a quantum phase estimation (QPE) circuit for the target Hamiltonian that operates on a 1D line with nearest neighbour gates; ii) Convert the circuit to one that acts on a 2D grid with nearest neighbour gates, and which is spatially sparse; iii) construct from this a spatially-sparse O(1)-local Hamiltonian that strongly simulates the original target Hamiltonian; iv) Apply a sequence of results from [CMP19] to construct a semi-translation-invariant simulator Hamiltonian on a 2D square lattice that proves the main result. The overhead (in terms of increase in number of particles, interaction strengths, and computation time) scales polynomially in the original number of qudits, and in particular the size of the circuit required to implement the QPE circuit.

Most of the work here goes into step iii): preparing a spatially sparse O(1)-local Hamiltonian that strongly simulates the target Hamiltonian, which can then be fed into the tools from [CMP19]. The main new ingredients for this are not hugely complicated —'uncomputing' and 'idling' (which has appeared in previous works) — however, there is some non-trivial work required to show that the resulting Hamiltonian simulates the target one.

For steps i) and ii), the authors make use of known results and tricks from the literature. Step iv) doesn't really use any new ideas, but does require careful application of the tools from [CMP19].

For the second result, the authors adapt an existing 1D Hamiltonian construction in order to apply their tricks of uncomputing and idling in that setting. The proof largely follows the previous one, except with that different circuit-to-Hamiltonian construction.

Summary

The paper is well written and mostly easy to follow, and I enjoyed reading it. The results are important ones, and a satisfying step forward for the field, building upon the groundwork laid by [CMP19] and follow-up works. Currently the results appear to be more theoretically interesting than practically so, although it is conceivable that these constructions could be used to do something useful with analog Hamiltonian simulation. That will heavily depend, however, on exactly how large the polynomial overhead is in the encodings used in the proofs. As the authors point out in the paper, there is already work showing the existence of strongly universal simulators for dynamics simulation, which could also be practically useful. Accompanying their observation of the differences between analog and dynamics simulation (see above), the authors claim that dynamics simulators would be less resilient to noise, and hint that they would be less useful in practice than a strong universal analog simulator. It would have been nice to see more discussion around this point, to build up some idea of why the authors think this to be the case.

The techniques to show the results are not particularly new, with the main insight being that one can construct a strong simulator via a Hamiltonian-to-circuit-to-spatially-sparse-Hamiltonian construction that avoids any sort of exponential blowup in resources. This uses a fairly standard clock construction from the Hamiltonian complexity literature, plus some additional tricks. The first part of the proof (constructing a spatially sparse NN circuit for QPE) is very straightforward, and I would argue doesn't require writing down a new proof beyond the purpose of self-containment of the paper (to be clear, I think the proof has a place in the paper, but mostly for reasons of presentation).

I would recommend acceptance of the paper. The results are interesting to a broad audience within the field of quantum computing and information, and I can see this paper serving as the go-to reference for universal Hamiltonian simulation results. The results are nicely presented, although (as discussed below) I think it would be nice if some of the presentations and proofs could be improved slightly.

Comments to authors

Main text:

Page 3, first column, Definition 1 — It would help if you specified exactly what spaces the V_i are isometries from and to (in fact you write that they act on one qudit from the original system, but my understanding is that these isometries act on a single qudit plus some 'local' ancilla space?).

Same place, more general comment — I actually found this definition much harder to parse than the original one in [CMP19]. Is there a reason that it differs to this extent? Either way it would be nice (although I understand there are space limitations)

to include a little bit of intuition or description of what these encodings are doing, for the reader unfamiliar with this topic.

Page 3, Definition 2 — Please define what ‘efficiently computable’ means wrt the description of H’.

Page 5, Claim 1 — I found the ‘time-independent’ statement confusing, when the encodings explicitly take a time parameter as input. I suppose the time-independence is that the encodings are the same at the beginning and end of the time evolution (but can still depend in some way on t?)

Page 7, second column, top: This paragraph and the one below it are verbatim repeats of some of the main text — is this intentional?

Page 9, Proposition 1 — Here you write ‘any n-qubit O(1)-local Hamiltonian’, but above you write ‘any digitally simulable Hamiltonian’. However there are digitally simulable Hamiltonians (e.g. O(log n)-local) that are not O(1)-local.

Same place, although it appears throughout — It wasn’t clear to me why the QPE circuits add an additional m ancilla qubits, but only use s of them to read off the eigenvalue. Why the extra m-s qubits in the first place? What are they used for?

Page 10, first column, first paragraph — This U_circuit is of course an identity operation in the end. Even with the preceding discussion, I found it quite surprising on a first read that this gives anything useful at all. I suppose the reason is that the temporal properties of the circuit (which are non-trivial) are encoded physically into the (time-independent) Hamiltonian, which is therefore non-trivial. It would be nice to see some mention of why the nature of the identity doesn’t lead to a trivial Hamiltonian.

Typos:

Page 3, first column, below Definition 2 — “all local A observables” should I think be “all local observables A”.

Page 5, first column, paragraph below section heading — “the distinction may appears negligible”, appears -> appear

Page 10, first column, second paragraph — “We then use perturbative argument..”: argument -> arguments

Supplementary information:

Page 2, above Eq. (3) — Perhaps I missed something, but the observation that exp(-i \mathcal{E}(H) t) has that form was not immediately obvious to me without thinking harder about the encodings. For the reader not too familiar with the encodings from [CMP19], perhaps a small note explaining this observation could be helpful?

Same place — Likewise, some clarification about the choice of state encoding would be useful. These things are not always very intuitive, and any extra help with the intuition would hugely improve the presentation of these proofs, I think.

Page 5, Section III A — I think the presentation of this section could be improved somewhat. The section begins by introducing lemmas 1 and 2, which refer to objects that aren’t defined until the proof of Proposition 2 (e.g. H_eff is not defined until eq (26), even though it is referenced in the statement of Lemma 2). This dislocation of definitions and statements and proofs made the section tricky to follow. Perhaps it would be good to describe the overall Hamiltonian construction before proceeding to prove anything?

Page 5, Proposition 2 — For qudit systems, is the dimension of the qudits assumed to be constant? I’m not sure this is explicitly addressed anywhere.

Page 8, below eq. (37) — You write “H_in penalizes the ancilla if it’s not in the state |0> before it is first used by the t_min(i)-th gate.” Is there a reason to construct H_in in such a way, and not use the usual construction that penalizes any computation that doesn’t begin (at time t=0) with the ancillas in the all-zeros state, and then leave it up to the propagation Hamiltonian to penalize deviations from this after time 0?

Page 9, Lemma 4 — Is V a local isometry here? (In the sense of Definition 1.)

Page 15, eq. (76) — I think the sum should range over t=0 to K+L ?

Typos:

Page 2, above eq. (3) — “state-encodings” -> state-encoding

Page 5, start of section III — “for strongly universal Hamiltonian in 2D square lattice” -> “for strongly universal Hamiltonians

on a 2D square lattice“

Page 6, just above eq. (23) — “To address these issue” -> “To address this issue”

Page 8, Lemma 3 — “be sum of” -> “be the sum of”

Page 10, end — “no any Y terms” -> “no Y terms”.

Page 11, top — “the gadget perform” -> “the gadget performs”

Page 11, paragraph below Lemma 9 — “converts term of the form” -> “converts terms of the form”

Page 12, below eq. (57) — “special boundary that separating” -> “special boundary that separates”

Page 15, eq. (79) — “gamma” -> “\gamma”

Version 1:

Reviewer comments:

Reviewer #1

(Remarks to the Author)
I thank the authors for preparing an updated version of the manuscript. My only substantial questions in the original review concerned the experimental realization of their construction, and the authors now address these questions in a satisfactory manner. I am happy for the paper to proceed to publication.

Reviewer #3

(Remarks to the Author)
Thanks to the authors for responding so thoroughly to my comments and those of the other referees on a previous version of this manuscript. I believe that their modifications to the manuscript have addressed the (minor) concerns I previously raised around the presentation of proofs, and their comments have cleared up the questions and misunderstandings that I had. I recommend acceptance of the current version of the manuscript to Nature Communications.

Open Access This Peer Review File is licensed under a Creative Commons Attribution 4.0 International License, which permits use, sharing, adaptation, distribution and reproduction in any medium or format, as long as you give appropriate credit to the original author(s) and the source, provide a link to the Creative Commons license, and indicate if changes were made.
In cases where reviewers are anonymous, credit should be given to 'Anonymous Referee' and the source.
The images or other third party material in this Peer Review File are included in the article’s Creative Commons license, unless indicated otherwise in a credit line to the material. If material is not included in the article’s Creative Commons

license and your intended use is not permitted by statutory regulation or exceeds the permitted use, you will need to obtain permission directly from the copyright holder.
To view a copy of this license, visit https://creativecommons.org/licenses/by/4.0/

Dear Reviewers,

We would like to thank you for the time and effort you invested in providing detailed and valuable evaluations of our manuscript. We have incorporated your feedback in the current version of our manuscript. Passages that have been revised substantially are highlighted in blue. We have also made minor changes throughout the entire manuscript. In what follows, we repeat your comments and suggestions in italics and address them one by one.

Reviewer #1 (Remarks to the Author)

The main result of this work is what the authors call a "strongly universal simulation" of O(1)-local Hamiltonians using local Hamiltonians on 2D and 1D grids. In the case of reduction to 2D, the resulting Hamiltonian can be composed of Heisenberg or XY interactions alone. In contrast to "weakly universal simulation" constructions given in previous works, the "strong" version has only a polynomial overhead in the number of particles or interaction energy. This has the conceptual significance of removing all exponential overheads from the simulation, which also brings it closer to experimental reality.

To construct their "strongly universal Hamiltonians", the authors draw from an arsenal of techniques in Hamiltonian complexity theory. The key first step is to consider the phase estimation circuit for the Hamiltonian which is the target for simulation. For O(1)-local Hamiltonians, such a circuit can then be brought down to a 2D or even 1D locally sparse form, using known techniques in complexity theory. The resulting circuit can in turn be mapped via a circuit-to-Hamiltonian construction to a locally sparse history Hamiltonian, with extra penalties for the phase-estimated energy. The authors prove that this locally-sparse Hamiltonian simulates the original one, penalty terms reproducing the spectrum of the original Hamiltonian. Conversion to a 2D square grid with a single type of interaction (e.g., Heisenberg), is done in the same way as in the earlier "weak Hamiltonian simulation" result by Cubitt, Montanaro and Piddock. Unlike in the latter work, the constructed Hamiltonian suffers no exponential overhead in energy, thanks to the history Hamiltonian constructed by the authors already being locally sparse in 2D. This also shows that the family of weakly universal Hamiltonians of Cubitt et al is in fact strongly universal.

In addition to the key result, the authors analyzed a variety of important auxiliary questions. For example, they pin down the precise relation between the subtly different concepts of "dynamics" and "analog" simulation. Also given is the proof of 2D strongly universal Hamiltonians for interactions of "non-2SLD" type, which generalizes the Heisenberg and XY options. Furthermore, the authors discuss possible extension of their results to fully translationary invariant Hamiltonians, and the implications of their work for simulations of Hamiltonians beyond O(1)-local (e.g., via quantum Church-Turing thesis).

The problem of "strong simulability" by 1D and 2D Hamiltonians, addressed by the authors, is significant conceptually as well as for experimental realizations of analog quantum simulation. The problem is nontrivial, and was even regarded as potentially unsolvable in the paper of Cubitt et al. To solve it, the authors had to draw on a variety of complexity-theoretic ideas in an original and technically sophisticated way. The authors' analysis

of dynamics vs. analog simulation and other auxiliary questions are also very welcome additions to the field. Finally, I find the very fact of successfully applying complexity-theoretic ideas to realistic analog quantum simulation a remarkable achievement in theoretical physics and computer science.

One minor thing I felt is missing is a concrete discussion about the possible next steps in making such an analog simulation realistic. Of course, a satisfactory analysis of the precise costs involved in implementing the authors' protocol would constitute a major work in itself; improving these costs further is a clearly promising line of future research. However, a brief discussion of these aspects would be a welcome addition to the field, given that many practically minded people are alien to complexity theoretic techniques. In particular, the reader may be interested in the authors' expert opinion on

1. *Which transformations in their construction should be revisited first, if one wants to improve the scaling with n and error, as well as the prefactor constants, in the particle count and prefactor energies? How much can it be improved? (I find it already useful that the authors comment on impossibility of attaining constant overhead in energies; having more of such insights in writing would be useful)*
2. *Which hardware may be best suited for simulation? (given the peculiarity of high energy overheads, high qubit counts, high control requirements, etc)*
3. *Which imperfections in hardware should one be most worried about? (e.g. stray or uncontrolled interactions - which strength, which type)*
4. *Which scaling of the prefactor energies may be satisfactory from realistic standpoint (if polynomial, then which degree, etc), and if there are any strong no-go statements about achieving practical analog simulation via such a universal reduction*
5. *Which trade-offs exist between different requirements on the simulating Hamiltonian, which are useful to keep in mind for applications? (e.g., if one is happy doing non-semi-TI physics, then one saves qubits and energy on mapping to Heisenberg; maybe some concrete families are cheaper to simulate than a general O(1)-local H; etc)*

Comments which would shed light on any of the above questions would benefit the paper and may greatly assist future research. I leave it up to the authors which exact points and to which extent they choose to elaborate on.

Author response: We thank the reviewer for this helpful suggestion to improve the manuscript. In response, we have greatly expanded the final paragraph in the Discussion section to include more practical outlook that addresses the requested points while remaining within the scope of the present work. Specifically, we identify the non-perturbative Hamiltonian-circuit-Hamiltonian mapping as the primary bottleneck in the resource scaling, and discuss possible avenues for improvements. We also commented on the suitability of superconducting qubit system, with native or programmable XY couplings for implementing our 2D universal simulators, and discussed the trade-off between enforcing semi-TI and reducing resource overheads. See below for the full paragraph of added discussion:

While this work constructs efficient universal analog quantum simulators using simple 1D or 2D systems, with only polynomial overheads in all resources, the constructions

presented here are not optimized for actual implementation, and there is much room for improvement for practical applications. The main contributor to the resource overhead in our analysis is the non-perturbative Hamiltonian-circuit-Hamiltonian pipeline, which is currently dominated by the Trotter decomposition of e^{-iHt} in the phase-estimation subroutine and amplified by the energy penalties involved in the circuit-Hamiltonian construction. These overheads could potentially be improved by replacing Trotterization with QSVT-based [34] method which has an exponentially better dependence on the precision parameter. Furthermore, we can consider a hybrid-approach that applies the costly non-pertubative mapping to only the high-degree or high-locality terms in the input Hamiltonian while handling the rest with cheaper perturbative gadgets. On the hardware side, superconducting qubit platforms [16] with native or programmable Heisenberg/XY couplings are well-suited to implement our 2D universal Hamiltonians; it would also be interesting to construct other universal families of Hamiltonians tailored to neutral atom and trapped ion platforms [35, 36] whose geometries are more flexible. More generally, there are useful trade-offs between structural constraints and resource costs; for example, relaxing semi-translation-invariance can reduce resource overheads by dispensing with gadgets that enforce a single interaction type. We further anticipate that leveraging other methods, such as the recently proposed dissipative gadgets [37], may achieve simulation with better resource scaling. As experimental realizations of analog quantum simulators develop rapidly, we hope our work helps to expand their scope to simulate all physical systems and tackle classically intractable problems.

We believe this additional discussion have shed light on questions (1), (2), and (5) posed by the reviewer, while making clear that a detailed resource-level optimization would constitute a substantial separate research effort, as the reviewer rightly notes.

By the way, please see a minor typo – "contains no any Y terms", supplement p. 10.

Author response: We have now corrected this typo.

To conclude, I enthusiastically recommend this paper for acceptance in Nature communications. Furthermore, I found only a very limited need for revisions (as described above). The manuscript is exceptionally well-written and well-structured, explaining the result on multiple levels of detail. Having carefully examined the work including the Supplemental Material p.II-IV, I found no logical flaw in the authors' argument. Most questions I had were answered in the text in a very clear way.

I sincerely congratulate the authors on attaining these great results, and express my gratitude as a reader for writing an excellent manuscript.

Reviewer #2 (Remarks to the Author)

Summary and main results: This paper is about analog simulations of quantum systems, which is where a quantum system is constructed so that its low energy space reproduces a different target system (up to some encoding). This is based on the definition from ref [16] which also showed that certain families of Hamiltonians are universal in the sense that they can simulate all other Hamiltonians. Previous constructions included qubit simulator Hamiltonians restricted to 2D grids [16] and were extended in [18,19] to

translationally invariant Hamiltonians in 2D and 1D. But the previous constructions with restricted spatial locality had interaction strengths that scaled exponentially with the size of the target system, and so were deemed inefficient.

The main contribution of this paper is efficient (ie poly sized interaction strengths) universal simulator constructions for 2D qubit and 1D qudit (8 dimensional subsystems) Hamiltonians, which are not translationally invariant. It is also shown that efficiently universal Hamiltonians can efficiently simulate any Hamiltonian whose time dynamics can be efficiently simulated. Finally there is a comparison to a different definition of dynamics simulation, and it is shown that the definition of analog simulation from [16] is stronger.

Correctness: The results all seem to be correct as far as I have checked them and are presented rigorously in the Supplementary Information, although I have not checked all of this. The detailed proof sketches in the Methods appendix are clear enough descriptions of the proofs (and how they work using previous methods combined in a logical way) that I have good confidence all of the results are correct.

Proof methods: The proofs of the constructions in 2D and 1D are both based on an idea from [21] by the same authors. It involves a very clever combination of standard quantum computing techniques: convert the target Hamiltonian into a circuit for dynamics simulation, then do phase estimation on this circuit, and convert back to a Hamiltonian using Kitaev's history state construction. Adding penalty terms to get the right energy for each state, and an idling trick to get a local encoding, completes the proof. Simple tricks for sparsifying the intermediate circuit results in a sparse simulator Hamiltonian. This powerful idea provides an easy way to generate new analog simulator constructions (by replacing the last step with a different circuit-to-Hamiltonian construction), and this crudely describes the approach taken in [19] and here.

There are a couple of extra tricks used here: for the 2D result, the circuit is transformed into a 1D nearest neighbour circuit, then using SWAP gates to a spatially sparse 2D circuit where the second dimension represents time. This results in a circuit which is spatially sparse so that the standard techniques of ref [27] can be used to convert to a 2D simulator Hamiltonian. For the 1D result, the circuit-to-Hamiltonian construction of [29] is used but here the idling trick would result in a highly non-local encoding. This is circumvented by a simple modification to the transition rules so that the idling can be done "in place".

To summarise my comments on proof methods: the main ideas largely come from other papers, but they are combined in a non-trivial way to get the genuinely new results, and there are other technical challenges which are neatly overcome with some simple tricks.

Significance of results: Analog simulators could be a practical use of small quantum computers or systems without error correction, and it is promising that previous work showed that simple qubit interactions can be universal. However for these simulators to be useful they need to be as close to practically realisable as possible. Previous constructions had the major drawback of either having interaction strengths growing exponentially with system size, or having long range interactions. The improvement here to efficient universal simulators in 2D therefore represents a major step forward in our understanding of analog simulators (with respect to the strong definition of [16]) and to their potential practical application. Overall I liked the results, and I found the paper clear and well-written, so I

would recommend acceptance.

Other comments: I realise the first draft of this paper appeared on the arXiv in Feb 2021, and the literature review is generally very good, but I'd be interested if the authors had some brief comments on this paper https://arxiv.org/abs/2101.12319. It came on the arXiv at about the same time (just before), and has some general conditions for analog simulations to be efficient and universal can these conditions be easily checked for the 2D and 1D cases in this paper?

Author response: We thank the reviewer for recognizing the significance of our work and alerting us to this highly relevant paper. We have carefully reviewed the paper and find that the general condition for efficient universal analog simulation they identified are compatible with the 2D and 1D families of Hamiltonian in our manuscript. To check that their condition holds, one will need a new proof that the 2D and 1D Hamiltonians are QMA-complete under faithful reduction; we believe this can be done by adopting arguments in our proof of Proposition 2 and Theorem 2, respectively. In this sense, the two papers are complementary.

To clarify the connection between these works for the reader, we have added the following paragraph to the Discussion section:

An independent concurrent work [33] proved that a family of Hamiltonians is strongly (or in their terminology, efficiently) universal if and only if it is closed under addition and QMA-complete under what they call faithful reductions. They showed that the circuit-to-Hamiltonian construction used in Ref. [27] to prove QMA-completeness of general local Hamiltonians (without restriction on geometry) can be modified to satisfy these conditions. Their proof strategy for showing faithfulness bears some similarities to our approach (e.g., idling trick). To check that their condition holds for the Hamiltonian families considered in the current paper, one would need a new proof that these 2D and 1D Hamiltonians are QMA-complete under faithful reduction; we believe that this can be done by adopting arguments in our proof of Proposition 2 and Theorem 2. This would provide a somewhat different proof of the strong universality of the Hamiltonian families in Theorems 1 and 2, respectively.

I am not convinced that you need to introduce the new definitions of "strong simulation" and "weak simulation", when the same idea (as I understand it) was previously covered by "efficient" and "inefficient". I realise that efficiency is very important for implementations of simulators, and that calling it strong/weak emphasises this point, but I feel introducing extra terminology may be unnecessarily confusing, so I'd ask the authors to consider sticking to efficient/inefficient?

Author response: We appreciate the reviewer's concern about potentially superfluous definitions. We had carefully considered adopting the "efficient/inefficient" dichotomy. However, we ultimately chose the "strong/weak" terminology because because it more precisely reflects the distinction relevant to this paper.

The key reason is that weakly universal families of Hamiltonians are not necessarily inefficient; rather, they are merely not shown to be efficient in all cases, and they still can be efficient for specific target Hamiltonians. For example, the proof of weak universality of 2D Hamiltonians in Ref. [17] already demonstrated efficient simulation for 2D target Hamiltonians. In our work, we further show (Theorem 1) that these same

weakly universal Hamiltonians ultimately also strongly universal, making it awkward to describe them as both "inefficient" and "efficient" universal simulator. Therefore, we find the "strong/weak" terminology better captures the disctinction between proven efficiency and currently unknown efficiency.

To clarify this choice and avoid confusion with existing terminology in the literature, we have added the following paragraph after Definition 3:

We remark that this notion of strong universality is equivalent to the "efficient universality" used in prior works (e.g., [17]). We adopt the "strong/weak" terminology here to emphasize that some universal families lack a known polynomial bound on resources. Note that this does not mean that they are provably inefficient.

Typo beneath equation (9) of supplementary information: implmentation.

Author response: We have now corrected this typo.

Reviewer #3 (Remarks to the Author)

Overview

This paper considers the notion of analog Hamiltonian simulation – that is, simulating, or reproducing the physics of, a target Hamiltonian by implementing another 'simulator' Hamiltonian. In a previous work, Cubit, Montanaro and Piddock [CMP19] formalized the theory of analog Hamiltonian simulation, and demonstrated the existence of 'universal' families of Hamiltonians – those that can simulate any target Hamiltonian. Remarkably, it turned out that many simple families of Hamiltonian, such as the Heisenberg or XY model on a square lattice, are instances of such universal Hamiltonians.

Such results are both theoretically interesting, having been used to prove various results in quantum complexity theory, as well as practically relevant: they open the door to simulating a wide range of quantum mechanical systems via the implementation of a chosen universal Hamiltonian, a use-case that covers one of the most important potential applications of quantum computers, and possibly without requiring a fault-tolerant gate-based device. Unfortunately, the aforementioned universality results came with a drawback: the simulations are only efficient for 1D and 2D target Hamiltonians. For more general target Hamiltonians, the simulators require resources growing exponentially with system size.

In this paper, the authors overcome this drawback, and demonstrate that the universal families of Hamiltonians considered in that work (and follow-up works), as well as a new 1D simulator Hamiltonian, can in fact simulate all target local Hamiltonians with only a polynomial increase in resources. They call this 'strong universal simulation' to distinguish it from the 'weak universal simulation' of previous results. This could give rise to new theoretical results (indeed a previous version of this paper has already yielded new results in some complexity theory works), as well as opening the door to efficient analog simulation of important Hamiltonians that are not 1D or 2D (such as the electronic structure Hamiltonians appearing in chemistry).

Results

*The authors use a different approach to previous works in this area to show that all Hamiltonians that are efficiently simulable via quantum circuits (in the sense of implementing time evolution) can be efficiently strongly simulated by a range of 2D and 1D families of Hamiltonians. The 2D families were already known (to be *weak* universal simulators), whereas the 1D family is new. Specifically, the main results shown in this paper are:*

*The existence of strongly universal families of 2D Hamiltonians. These are so-called semi-translationally invariant (meaning that the interactive *terms* in the Hamiltonian are identical across sites, but the interaction *strengths* are not). This is in contrast to some known (fully-)translationally invariant weakly universal families, though one probably cannot expect to recover full translation-invariance for strong universality. The existence of a strongly universal family of 1D Hamiltonians (on 8-dimensional audits, not (semi-)translationally invariant).*

The authors also consider the notion of dynamics simulation: using the time-dynamics of a simulator Hamiltonian to simulate the time-dynamics of a target Hamiltonian. The authors show that analog simulation (in the sense defined by [CMP19]) is a strictly stronger condition than dynamics simulation. In particular, it is shown that every analog simulator is a 'dynamics' simulator (with some appropriate definition thereof), but that the converse does not necessarily always (fully) hold. Strong universality for dynamics simulation has been shown in previous works, and so this fact separates the contribution of the current work with those.

Techniques

For the first result, the main steps of the proof are i) to construct a quantum phase estimation (QPE) circuit for the target Hamiltonian that operates on a 1D line with nearest neighbour gates; ii) Convert the circuit to one that acts on a 2D grid with nearest neighbour gates, and which is spatially sparse; iii) construct from this a spatially-sparse O(1)-local Hamiltonian that strongly simulates the original target Hamiltonian; iv) Apply a sequence of results from [CMP19] to construct a semi-translation-invariant simulator Hamiltonian on a 2D square lattice that proves the main result. The overhead (in terms of increase in number of particles, interaction strengths, and computation time) scales polynomially in the original number of qudits, and in particular the size of the circuit required to implement the QPE circuit.

Most of the work here goes into step iii): preparing a spatially sparse O(1)-local Hamiltonian that strongly simulates the target Hamiltonian, which can then be fed into the tools from [CMP19]. The main new ingredients for this are not hugely complicated – 'uncomputing' and 'idling' (which has appeared in previous works) – however, there is some non-trivial work required to show that the resulting Hamiltonian simulates the target one.

For steps i) and ii), the authors make use of known results and tricks from the literature. Step iv) doesn't really use any new ideas, but does require careful application of the tools from [CMP19].

For the second result, the authors adapt an existing 1D Hamiltonian construction in order to apply their tricks of uncomputing and idling in that setting. The proof largely follows the previous one, except with that different circuit-to-Hamiltonian construction.

Summary

The paper is well written and mostly easy to follow, and I enjoyed reading it. The results are important ones, and a satisfying step forward for the field, building upon the groundwork laid by [CMP19] and follow-up works. Currently the results appear to be more theoretically interesting than practically so, although it is conceivable that these constructions could be used to do something useful with analog Hamiltonian simulation. That will heavily depend, however, on exactly how large the polynomial overhead is in the encodings used in the proofs. As the authors point out in the paper, there is already work showing the existence of strongly universal simulators for dynamics simulation, which could also be practically useful. Accompanying their observation of the differences between analog and dynamics simulation (see above), the authors claim that dynamics simulators would be less resilient to noise, and hint that they would be less useful in practice than a strong universal analog simulator. It would have been nice to see more discussion around this point, to build up some idea of why the authors think this to be the case.

Author response: We thank the reviewer for affirming the importance and readability of our result. While our current result indeed focuses on establishing a theoretical foundation for efficient universal Hamiltonian simulator, we refer to our response to Reviewer 1 on the possible avenues to improve the practicality of our constructions.

To address the reviewer's specific question about our claim that dynamics simulator would be less resilient to noise, we have added the following discussion after Claim 1 to clarify our reasoning:

A dynamics simulator need not function as an analog simulator of the full Hamiltonian because it can possess unwanted low-energy eigenstates without counterparts in the target system. An example is illustrated in Fig. 1. Such a dynamics simulator only becomes an analog simulator after restriction to the desired eigenstates; however, any uncontrolled leakage into the unwanted eigenstates could cause the analog simulation to fail. In particular, thermal fluctuations and other noise sources can drive the dynamics simulator to populate these low-energy eigenstates, preventing it from faithfully reproducing the equilibrium thermodynamical properties of the target Hamiltonian. Moreover, a dynamics simulator may allow a nonlinear time parametrization $t' = f(t)$ that can distort the spectrum significantly and further obstruct faithful analog simulation.

The techniques to show the results are not particularly new, with the main insight being that one can construct a strong simulator via a Hamiltonian-to-circuit-to-spatially-sparse-Hamiltonian construction that avoids any sort of exponential blowup in resources. This uses a fairly standard clock construction from the Hamiltonian complexity literature, plus some additional tricks. The first part of the proof (constructing a spatially sparse NN circuit for QPE) is very straightforward, and I would argue doesn't require writing down a new proof beyond the purpose of self-containment of the paper (to be clear, I think the proof has a place in the paper, but mostly for reasons of presentation).

I would recommend acceptance of the paper. The results are interesting to a broad audience within the field of quantum computing and information, and I can see this paper serving as the go-to reference for universal Hamiltonian simulation results. The results are nicely presented, although (as discussed below) I think it would be nice if some of the presentations and proofs could be improved slightly.

Author response: We thank the reviewer for a positive recommendation for the paper. For the minor issues on presentation and proofs noted by the reviewer, we address them

point-by-point below.

Comments to authors

Main text:

Page 3, first column, Definition 1 – It would help if you specified exactly what spaces the V_i are isometries from and to (in fact you write that they act on one qudit from the original system, but my understanding is that these isometries act on a single qudit plus some 'local' ancilla space?).

Same place, more general comment – I actually found this definition much harder to parse than the original one in [CMP19]. Is there a reason that it differs to this extent? Either way it would be nice (although I understand there are space limitations) to include a little bit of intuition or description of what these encodings are doing, for the reader unfamiliar with this topic.

Author response: Here, $V : \mathbb{C}^d \to (\mathbb{C}^{d'})^{\otimes k}$ is an isometry that maps a single qudit in the original system to a group of k qudits in the simulator, for some unspecified positive integer k such that $d \leq (d')^k$. We wanted to avoid the unnecessary introduction of an extra symbol k, and hence simply states that "V_i is an isometry that acts on one qudit in the original system". Note this is very similar to the wording in the definition by [CMP19] that states "V_i acting on 0 or 1 qudits of the original system each".

It is possible that our definition here is more complex than the original one in [CMP19] because we strive to include self-contained definition of all non-standard terminology. For example, the term "locally orthogonal projectors" is not explicitly defined in the definition in [CMP19] until much later, whereas we define it explicitly in Definition 1.

Page 3, Definition 2 – Please define what 'efficiently computable' means wrt the description of H'.

Author response: Thank you for pointing this possible point of confusion. We have revised it to say:

H'_m is efficiently computable in $O(\text{poly}(n, \eta^{-1}, \epsilon^{-1}, \Delta))$ time from the description of H.

Page 5, Claim 1 – I found the 'time-independent' statement confusing, when the encodings explicitly take a time parameter as input. I suppose the time-independence is that the encodings are the same at the beginning and end of the time evolution (but can still depend in some way on t?)

Author response: The statement $\mathcal{E}^{\text{state}}_{\text{in}}(\rho, t) = \mathcal{E}^{\text{state}}_{\text{out}}(\rho, t) = V\rho V^\dagger$ in Claim 1 is used to indicate that both $\mathcal{E}^{\text{state}}_{\text{in}}(\rho, t)$ and $\mathcal{E}^{\text{state}}_{\text{out}}(\rho, t)$ have no t-dependence (since V has no t-independence) and that they are equal. To improve clarity, we have modified the statement to read:

$\mathcal{E}^{\text{state}}_{\text{in}}(\rho, t) = \mathcal{E}^{\text{state}}_{\text{out}}(\rho, t) = \mathcal{E}^{\text{state}}(\rho)$.

Page 7, second column, top: This paragraph and the one below it are verbatim repeats of some of the main text – is this intentional?

Author response: We thank the reviewer for pointing out the redundancy. We have revised the beginning of the Methods section to reduce unnecessary repetition. It now reads:

The starting point of our proofs for both Theorem 1 and 2 is as follows. We note that previous constructions [1719, 28] relied purely on perturbative gadgets to reduce the connectivity of qudits. As mentioned earlier in the Results section, reducing the degree in the interaction graph from $\Omega(n)$ to $O(1)$ required $\Omega(\log n)$ rounds of perturbative gadget, yielding a final Hamiltonian with interaction energy blowing up as $2^{\Omega(\mathrm{poly}(n))}$.

Instead, we reduce the connectivity of the qudits in our construction by first mapping the target Hamiltonian H to a quantum circuit performing the phase estimation algorithm with respect to e^{-iHt}. Using standard techniques including Trotter decomposition [38], we can embed this circuit in 1D, using only nearest-neighbor gates on a line of qubits, while still applying the desired phase estimation with sufficient accuracy....

Page 9, Proposition 1 – Here you write 'any n-qubit O(1)-local Hamiltonian', but above you write 'any digitally simulable Hamiltonian'. However there are digitally simulable Hamiltonians (e.g. O(log n)-local) that are not O(1)-local.

Author response: We thank the reviewer for pointing out this inconsistency. In fact, we mean for Proposition 1 to apply to any digitally simulable Hamiltonian, and have revised both the manuscript and the Supplementary Information to reflect this.

Same place, although it appears throughout – It wasn't clear to me why the QPE circuits add an additional m ancilla qubits, but only use s of them to read off the eigenvalue. Why the extra $m - s$ qubits in the first place? What are they used for?

Author response: The extra $m-s$ qubits are used as additional workspace, as needed, to faithfully simulate QPE with s-bit precision. For example, the analysis of QPE in [Nielsen and Chuang, 2011], which we restate in Supplementary Eq. (12), indicates that more ancilla qubits help reduce errors in the QPE results.

Page 10, first column, first paragraph – This U_{circuit} is of course an identity operation in the end. Even with the preceding discussion, I found it quite surprising on a first read that this gives anything useful at all. I suppose the reason is that the temporal properties of the circuit (which are non-trivial) are encoded physically into the (time-independent) Hamiltonian, which is therefore non-trivial. It would be nice to see some mention of why the nature of the identity doesn't lead to a trivial Hamiltonian.

Author response: We thank the reviewer for highlighting this counterintuitive fact. To emphasize the importance of the temporal property of U_{circuit}, we have added the following explanatory sentence:

Although $U_{\mathrm{circuit}} = \mathbb{I}$ is ultimately a trivial operation, the circuit's nontrivial temporal structure allows its computational history to encode the spectral information about the target Hamiltonian.

Typos:

Page 3, first column, below Definition 2 – "all local A observables" should I think be "all local observables A".

Page 5, first column, paragraph below section heading – "the distinction may appears negligible", appears → appear

Page 10, first column, second paragraph – "We then use perturbative argument..": argument → arguments

Author response: We have corrected all typos mentioned.

Supplementary information:

Page 2, above Eq. (3) – Perhaps I missed something, but the observation that $\exp(-i\mathcal{E}(H)t)$ *has that form was not immediately obvious to me without thinking harder about the encodings. For the reader not too familiar with the encodings from [CMP19], perhaps a small note explaining this observation could be helpful?*

Same place – Likewise, some clarification about the choice of state encoding would be useful. These things are not always very intuitive, and any extra help with the intuition would hugely improve the presentation of these proofs, I think.

Author response: We have added two more clarifying equations, in new Supplementary Equations (3) and (4), to explain the origin of those identities.

Page 5, Section III A – I think the presentation of this section could be improved somewhat. The section begins by introducing lemmas 1 and 2, which refer to objects that aren't defined until the proof of Proposition 2 (e.g. H_{eff} *is not defined until eq (26), even though it is referenced in the statement of Lemma 2). This dislocation of definitions and statements and proofs made the section tricky to follow. Perhaps it would be good to describe the overall Hamiltonian construction before proceeding to prove anything?*

Author response: The statement of Lemma 1 does not require H_{out} to be explicitly, only that it satisfies Eq. (21). The explicit form of H_{out} in Eq. (26) is just one of many possible choices. Furthermore, H_{eff} is already well-defined in the statement of Lemma 2. We have added a few clarifying words in the lemma statements and ensured that all objects are well-defined.

Page 5, Proposition 2 – For qudit systems, is the dimension of the qudits assumed to be constant? I'm not sure this is explicitly addressed anywhere.

Author response: Indeed, the dimension of the qudit is assumed to be constant, as implicit in the rest of the paper. To avoid confusion, we have added "$d = O(1)$" to the statement of Proposition 2.

Page 8, below eq. (37) – You write "H_{in} *penalizes the ancilla if it's not in the state* $|0\rangle$ *before it is first used by the* $t_{\min}(i)$*-th gate." Is there a reason to construct* H_in *in such a way, and not use the usual construction that penalizes any computation that doesn't begin (at time t=0) with the ancillas in the all-zeros state, and then leave it up to the propagation Hamiltonian to penalize deviations from this after time 0?*

Author response: Yes, the reason is to keep H_{circuit} spatially sparse. If H_{in} directly checks that all the ancilla qudits are in $|0\rangle$ at $t = 0$, then the clock qubit corresponding to $t = 0$ will interact with all m ancilla qudits, causing a potentially high degree node in the interaction graph that prevents spatial sparsity. Instead, our construction penalizes each ancilla qudit i when it's first used at $t = t_{\min}(i)$, so that no clock qubit is overwhelmed with many interactions. Since the propagation Hamiltonian ensures each qudit is unchanged before it is used, this construction also retroactively guarantees that the qudit is in state $|0\rangle$ at $t = 0$.

Page 9, Lemma 4 – Is V a local isometry here? (In the sense of Definition 1.)

Author response: No, it is not necessary for the isometry V to be local for the statement of Lemma 4 to be true. Consequently, we kept the more general condition.

Page 15, eq. (76) – I think the sum should range over t=0 to K+L ?

Author response: Yes, thank you for pointing out this typo. We have corrected it.

Typos:

Page 2, above eq. (3) – "state-encodings" → state-encoding

Page 5, start of section III – "for strongly universal Hamiltonian in 2D square lattice" → "for strongly universal Hamiltonians on a 2D square lattice"

Page 6, just above eq. (23) – "To address these issue" → "To address this issue"

Page 8, Lemma 3 – "be sum of" → "be the sum of"

Page 10, end – "no any Y terms" → "no Y terms".

Page 11, top – "the gadget perform" → "the gadget performs"

Page 11, paragraph below Lemma 9 – "converts term of the form" → "converts terms of the form"

Page 12, below eq. (57) – "special boundary that separating" → "special boundary that separates"

Page 15, eq. (79) – "gamma" → "γ"

Author response: We thank the reviewer for carefully reading our paper and catching these typos, and have corrected all of them as suggested.

Sincerely,

Leo Zhou and Dorit Aharonov
